# Cave-adapted beetles from continental Portugal

**DOI:** 10.3897/BDJ.9.e67426

**Published:** 2021-08-20

**Authors:** Ana Sofia P. S. Reboleira, Rita P. Eusébio

**Affiliations:** 1 Natural History Museum of Denmark, University of Copenhagen, Copenhagen, Denmark Natural History Museum of Denmark, University of Copenhagen Copenhagen Denmark; 2 Departamento de Biologia Animal, Faculdade de Ciências, University of Lisbon, Lisbon, Portugal Departamento de Biologia Animal, Faculdade de Ciências, University of Lisbon Lisbon Portugal; 3 Centre for Ecology, Evolution and Environmental Changes (cE3c), Faculdade de Ciências, University of Lisbon, Lisbon, Portugal Centre for Ecology, Evolution and Environmental Changes (cE3c), Faculdade de Ciências, University of Lisbon Lisbon Portugal

**Keywords:** Coleoptera, subterranean habitats, Iberian Peninsula, conservation, rocky habitats, troglobiont, stygobiont

## Abstract

**Background:**

The cave biodiversity of continental Portugal faces tremendous conservation challenges, mostly linked to their direct destruction and contamination infiltrating from the surface. Beetles are the most diverse insects and one of the most diverse arthropod groups in caves of Portugal.

**New information:**

We present the IUCN Red List profiles for the cave-adapted beetles from continental Portugal, all endemic to their respective geological units and massifs. Ground beetles (Carabidae) are the most diverse family of cave-adapted beetles in continental Portugal, followed by rove beetles (Staphylinidae). Beetles in caves of Portugal are mostly terrestrial and only one species is known to have evolved to live in groundwater. *Trechus* is the most diverse genus with four species, followed by *Domene* with three species and by *Speonemadus* and *Iberoporus*, both with one cave-adapted species. The aim of this contribution is to assess all endemic cave-adapted species of beetles from continental Portugal and to support their specific protection, to promote adequate management of surface habitats and the establishment of priority areas for conservation. The main biodiversity erosion drivers that are impacting the conservation of the studied species are pollution infiltrating from the surface, urbaniation, modifications of the natural habitat for touristic purposes and mining, quarrying and energy production infrastructures.

This document can be used in spatial planning and territory management in karst, based on the current scientific knowledge.

## Introduction

Cave fauna has relatively low specific richness, but high conservation value for the global biodiversity of our planet (Mammola et al. 2019). Subterranean species have unique traits that emerged as a result of isolation and convergent adaptation towards the underground life (Mammola et al. 2020).

Amongst subterranean fauna, the beetles (Insecta, Coleoptera) stand out, as a group that presents the greatest animal specific richness worldwide (Didham et al. 2020). Of the 211 recognised families of Coleoptera (Bouchard et al. 2011), around 10 have cave-adapted species, of which about 92% belong to the families Carabidae (mostly Trechinae) and Leiodidae (mostly Cholevinae) (Deharveng and Bedos 2018).

Portugal is located in the western part of the Iberian Peninsula, has more than 2000 caves identified and is considered a hotspot of subterranean biodiversity (Reboleira et al. 2011b). More than 58 terrestrial and 64 aquatic subterranean-obligate species are described to date (Reboleira et al. 2013a, Reboleira and Enghoff 2018, Ribera and Reboleira 2019). Beetles are the third most diverse order of terrestrial cave-adapted species in continental Portugal, preceeded by pseudoscorpions (13 species) and terrestrial isopods (12 species) (Reboleira et al. 2015, Zaragoza and Reboleira 2018).

The first beetle species, collected in caves of Portugal, was identified as *Trechusfulvus* Dejean, 1831, captured by M.L.W. Schaufuss; the caves then explored remain unknown (Putzeys 1870). The first cave-adapted beetle of Portugal, *Trechusmachadoi* Jeannel, 1941, was found more than a century later and described, based on two specimens collected by the pioneer of Portuguese cave biology, António de Barros Machado (Jeannel 1941). Since this time, almost seven decades have passed until the description of further cave-adapted beetles in continental Portugal, also belonging to the genus *Trechus* Clairville, 1806 (Reboleira et al. 2010). The first Staphylinidae beetle was described in 2010 from a karst cave, belonging to the genus *Domene* Fauvel, 1873 (Reboleira et al. 2011a), followed more recently by the description of two more species of the same genus from non-karst caves (Serrano et al. 2015, Magrini and Carotti 2019). Despite the high richness of Leiodidae in neighbouring Spain (Salgado et al. 2008), only one cave-adapted leiodid is known from Portugal (Reboleira et al. 2017).

All cave-adapted beetle species from continental Portugal are endemic and exhibit an extremely reduced distribution area. These unique species face major conservation threats and lack of adequate specific management (Reboleira et al. 2011b). Here, we present the IUCN Red List profiles for the nine species of cave-adapted beetles from continental Portugal.

## Material and Methods

Caves of continental Portugal have been intensively sampled over the last 15 years. Sampling was performed by direct searching and baited pitfall traps, both in caves and in the mesovoid shallow substratum. Caves consist of underground spaces where a human can fit, while the mesovoid shallow substratum (MSS) consists of a matrix of unaggregated rock that can be found in scree slopes. Most of this sampling was performed under the framework of Master and Doctoral studies (Reboleira 2007, Reboleira 2012, Eusébio 2020) and all specimens were collected under legal permits of the Instituto de Conservação da Natureza e das Florestas. Specimens were sorted and identified to species level using dissection and microscopy support and compared with collection specimens and the appropriate bibliography.

Extent of occurence (EOO) and area of occupancy (AOO) were calculated using the Geospatial Conservation Assessment Tool (GeoCAT) with an approximation to the standard IUCN 2 km × 2 km cells (4 km^2^) and the maps were created in the open source software QGIS 3.14.16, with the natural protected areas of Portugal layer (ICNF 2020).

Threats were observed *in situ* in the field and complemented by appropriate literature surveys. Threats, conservation actions, research needed and the type of habitat classification were assigned, based on the IUCN Red List database.

## Species Conservation Profiles

### Iberoporus pluto

#### Species information

Scientific name: Iberoporuspluto

Species authority: Ribera & Reboleira, 2019

Kingdom: Animalia

Phylum: Arthropoda

Class: Insecta

Order: Coleoptera

Family: Dytiscidae

Taxonomic notes: *Iberoporuspluto* is the largest and widest of all the subterranean species of its genus (Ribera and Reboleira 2019).

**Figure 1.**
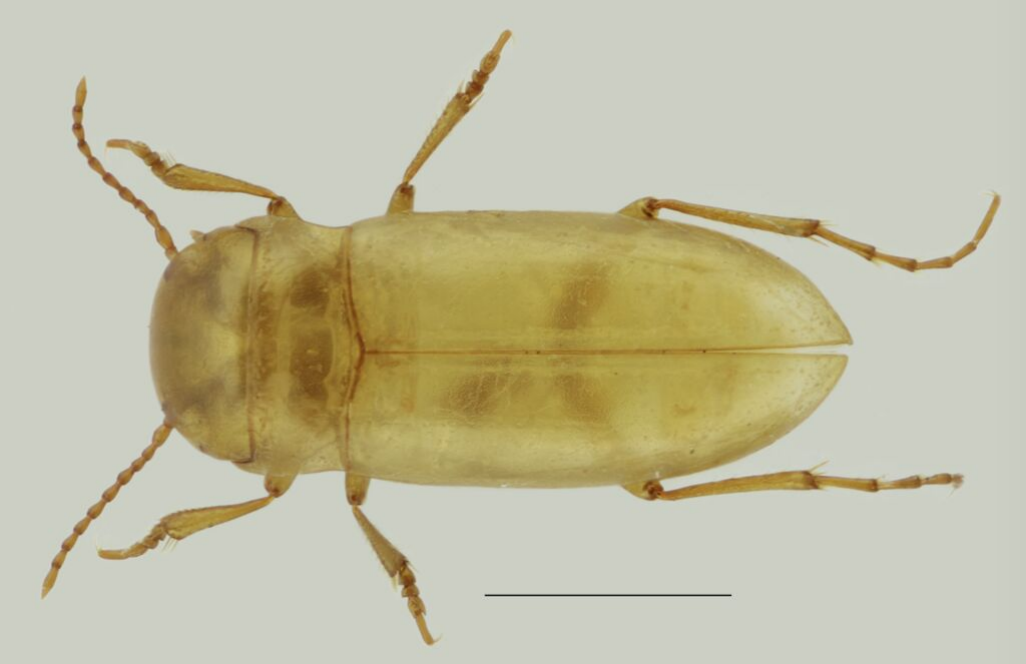
*Iberoporuspluto* Ribera & Reboleira, 2019. Soprador do Carvalho Cave, Penela, Coimbra District, Portugal. Scale bar: 1 mm.

Figure(s) or Photo(s): Fig. 1

Region for assessment: Europe

#### Geographic range

Biogeographic realm: Palearctic

Countries: Portugal

Map of records (Google Earth): Suppl. materials 1, 2

Basis of EOO and AOO: Known habitat extent

Basis (narrative): The extent of occurrence (EOO) and area of occupancy (AOO) are both 4 km².

Min Elevation/Depth (m): 218

Range description: *Iberoporuspluto* is a groundwater-adapted beetle known from a stream in a single cave, located in north-eastern Sicó karst area. The cave stream, where it was found, flows in a subterranean system of approximately 15 km of horizontal implementation (Ribera and Reboleira 2019).

#### New occurrences

#### Extent of occurrence

EOO (km2): 4

Trend: Decline (observed)

Justification for trend: A decline in EOO is inferred due to the degradation of the cave by anthropogenic impact as Soprador do Carvalho Cave is subject to recreational visitation and direct trampling on the subterranean stream.

Causes ceased?: No

Causes understood?: Yes

Causes reversible?: Yes

Extreme fluctuations?: Unknown

#### Area of occupancy

Trend: Decline (projected)

Justification for trend: No decline in AOO has been observed, but it is inferred due to the decline and vulnerability of the habitat.

Causes ceased?: No

Causes understood?: Yes

Causes reversible?: Unknown

Extreme fluctuations?: Unknown

AOO (km2): 4

#### Locations

Number of locations: 1

Justification for number of locations: *Iberoporuspluto* occurs in a single cave, Soprador do Carvalho, in the Sicó karst area in central Portugal and it is under moderate disturbance (Ribera and Reboleira 2019).

Trend: Stable

Justification for trend: This cave is the only known location in the historical range, as several caves in the same karst massif nearby have been sampled and this species has never been found elsewhere. Therefore, the current trend in number of locations is stable.

Extreme fluctuations?: Unknown

#### Population

Number of individuals: Unknown

Trend: Unknown

Causes ceased?: Unknown

Causes understood?: Unknown

Causes reversible?: Unknown

Extreme fluctuations?: Unknown

Population Information (Narrative): Only one specimen (female) of this species is known from a single location in central Portugal.

#### Subpopulations

Trend: Unknown

Extreme fluctuations?: Unknown

Severe fragmentation?: Unknown

#### Habitat

System: Freshwater

Habitat specialist: Yes

Habitat (narrative): The specimen was collected in the bottom of a clay pool connected to a subterranean stream (Ribera and Reboleira 2019).

Trend in extent, area or quality?: Decline (observed)

Justification for trend: Soprador do Carvalho Cave is located in the vicinity of a village, agricultural fields and a quarry (NEC 2020, Neves et al. 2005). It is also affected by touristic activities as tourists visit the cave and step on the stream (Ribera and Reboleira 2019).

##### Habitat

Habitat importance: Major Importance

Habitats: 5.1. Wetlands (inland) - Permanent Rivers/Streams/Creeks (includes waterfalls)7.1. Caves and Subterranean Habitats (non-aquatic) - Caves

#### Habitat

Habitat importance: Major Importance

Habitats: 5.1. Wetlands (inland) - Permanent Rivers/Streams/Creeks (includes waterfalls)7.1. Caves and Subterranean Habitats (non-aquatic) - Caves

#### Ecology

Size: 2.8 mm

Generation length (yr): 1

Dependency of single sp?: Unknown

Ecology and traits (narrative): The genus *Iberoporus* Castro & Delgado, 2001 is endemic to the Iberian Peninsula and exclusively stygobiont (Castro and Delgado 2001, Ribera 2003, Ribera and Reboleira 2019, Ribera et al. 2002). *Iberoporuspluto* has extreme troglomorphic traits (eyeless and depigmentation) and unusually elongated legs not adapted for swimming (Ribera and Reboleira 2019).

#### Threats

Justification for threats: The cave is explored for tourism and visitors step over the habitat where the species was found (Ribera and Reboleira 2019). Other threats include the proximity to a quarry located at 1.4 km from the cave entrance. It is also 67 m from the closest houses and is surrounded by agricultural lands. The subterranean stream flows below habitational areas and run-off of urban wastewater directly to the stream is observable (Reboleira et al. 2011b).

##### Threats

Threat type: Ongoing

Threats: 1.1. Residential & commercial development - Housing & urban areas3.2. Energy production & mining - Mining & quarrying6.1. Human intrusions & disturbance - Recreational activities9.1.2. Pollution - Domestic & urban waste water - Run-off

#### Threats

Threat type: Ongoing

Threats: 1.1. Residential & commercial development - Housing & urban areas3.2. Energy production & mining - Mining & quarrying6.1. Human intrusions & disturbance - Recreational activities9.1.2. Pollution - Domestic & urban waste water - Run-off

#### Conservation

Justification for conservation actions: Measures should be taken to prevent infiltration of wastewaters from the village into the cave stream. The nearby quarry has been reported in the national media to be the source of the infiltration of small particles of quarry dust that have been deposited all over the gallery of Algarinho Cave by flood events. This type of slurry is known to perniciously impact groundwater quality (Piccini et al. 2019) and Algarinho Cave is part of the Dueça Speleological System, therefore, hydrologically connected to Soprador do Carvalho Cave.

##### Conservation actions

Conservation action type: Needed

Conservation actions: 1.1. Land/water protection - Site/area protection2.1. Land/water management - Site/area management2.3. Land/water management - Habitat & natural process restoration4. Education & awareness5.1.3. Law & policy - Legislation - Sub-national level

#### Conservation actions

Conservation action type: Needed

Conservation actions: 1.1. Land/water protection - Site/area protection2.1. Land/water management - Site/area management2.3. Land/water management - Habitat & natural process restoration4. Education & awareness5.1.3. Law & policy - Legislation - Sub-national level

#### Other

##### Use and trade

Use type: International

##### Ecosystem services

Ecosystem service type: Very important

##### Research needed

Research needed: 1.2. Research - Population size, distribution & trends1.3. Research - Life history & ecology2.2. Conservation Planning - Area-based Management Plan3.1. Monitoring - Population trends3.4. Monitoring - Habitat trends

Justification for research needed: Further investigation is needed about the distribution, ecology and life cycle of the species. Developing a management plan for this species is crucial. This plan will aid the conservation of the cave-adapted species of the Sicó karst area.

#### Use and trade

Use type: International

#### Ecosystem services

Ecosystem service type: Very important

#### Research needed

Research needed: 1.2. Research - Population size, distribution & trends1.3. Research - Life history & ecology2.2. Conservation Planning - Area-based Management Plan3.1. Monitoring - Population trends3.4. Monitoring - Habitat trends

Justification for research needed: Further investigation is needed about the distribution, ecology and life cycle of the species. Developing a management plan for this species is crucial. This plan will aid the conservation of the cave-adapted species of the Sicó karst area.

#### Viability analysis

### Trechus machadoi

#### Species information

Scientific name: Trechusmachadoi

Species authority: Jeannel, 1941

Kingdom: Animalia

Phylum: Arthropoda

Class: Insecta

Order: Coleoptera

Family: Carabidae

Taxonomic notes: This species belongs to the *T.fulvus*-group of species.

**Figure 2.**
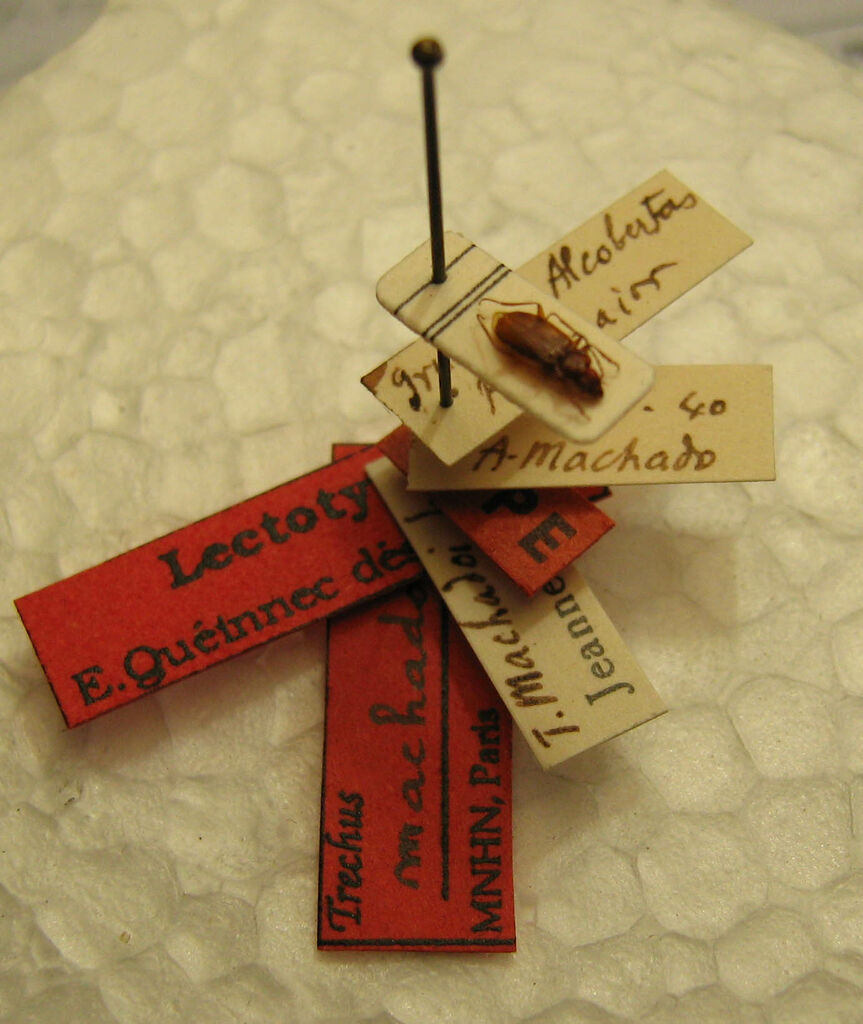
*Trechusmachadoi* Jeannel, 1941. Alcobertas Cave, Rio Maior, Portugal. Lectotype deposited at the Natural History Museum of Paris.

Figure(s) or Photo(s): Fig. 2

Region for assessment: Europe

#### Geographic range

Biogeographic realm: Palearctic

Countries: Portugal

Map of records (Google Earth): Suppl. materials 1, 3

Basis of EOO and AOO: Known habitat extent

Basis (narrative): The extent of occurrence (EOO) and the maximum estimated area of occupancy (AOO) are both 4 km^2^.

Min Elevation/Depth (m): 389

Max Elevation/Depth (m): 400

Range description: *Trechusmachadoi* is a troglobiont beetle known only in the Alcobertas Cave and in a countinuous mesovoid shallow substratum (MSS) located in the Serra dos Candeeiros subunit of the Estremenho karst massif, central Portugal. The cave extends horizontally for approximately 210 m (Reboleira 2007, Reboleira 2012, Reboleira et al. 2009) and the MSS area is located 80 m from the type locality (Eusébio 2020).

#### New occurrences

#### Extent of occurrence

EOO (km2): 4

Trend: Decline (observed)

Justification for trend: Despite intensive sampling in the type locality (cave), no specimens have been found in there since the species description. Only recent sampling in the MSS contiguous to the cave retrieved specimens.

Causes ceased?: Unknown

Causes understood?: Unknown

Causes reversible?: Unknown

Extreme fluctuations?: Unknown

#### Area of occupancy

Trend: Decline (observed)

Justification for trend: AOO decline has been inferred due to the vulnerability of the habitat.

Causes ceased?: Unknown

Causes understood?: Unknown

Causes reversible?: Unknown

Extreme fluctuations?: Unknown

AOO (km2): 4

#### Locations

Number of locations: 2

Justification for number of locations: *Trechusmachadoi* is known only from the Alcobertas Cave and from the adjacent MSS, located approximately 80 m from each other. Its distribution is likely to be confined to the subterranean habitats of the Serra dos Candeeiros subunit (Reboleira et al. 2009). This population is under intense disturbance.

Trend: Stable

Justification for trend: Alcobertas is the only known location in the historical range, therefore, the current trend in number of locations is stable.

Extreme fluctuations?: Unknown

#### Population

Number of individuals: Unknown

Trend: Unknown

Justification for trend: The type locality was monitored for six months in 2009, but no specimens were sampled or observed, most likely due to the human impact on this cave described below (Reboleira 2007).

Causes ceased?: No

Causes understood?: Yes

Causes reversible?: Unknown

Extreme fluctuations?: Unknown

Population Information (Narrative): So far, only one population is known from the Alcobertas Cave, which is also dispersed in the contiguous mesovoid shallow substratum at 0.5 m depth in scree slopes (Reboleira 2007, Reboleira et al. 2009, Reboleira et al. 2010).

#### Subpopulations

Trend: Unknown

Extreme fluctuations?: Unknown

Severe fragmentation?: No

#### Habitat

System: Terrestrial

Habitat specialist: Yes

Habitat (narrative): The Alcobertas Cave was subject of a large anthropogenic intervention at the beginning of the 1970s, with the intention to transform it to receive mass tourism. During that process, a second entry was opened near the end of the gallery which induced significant changes in the climatology of the cave (Reboleira 2007). Many explosions inside the cave have resulted in large accumulations of powdery residues that can still be observed nowadays, which, associated with the air fluxes between the two entrances, may have displaced the *Trechusmachadoi* population to other parts of the subterranean network (Reboleira et al. 2009). The Alcobertas Cave is currently used for tourism by a local association. Recently, *Trechusmachadoi* was collected during winter at a depth of 50 cm in the mesovoid shallow substratum (MSS) in scree slopes close to the type locality (Eusébio 2020, Eusébio et al. 2021). These scree slopes are classified as western Mediterranean and thermophile deposits and protected by the Natura 2000 network (Eusébio et al. 2021).

Trend in extent, area or quality?: Decline (inferred)

##### Habitat

Habitat importance: Major Importance

Habitats: 7.1. Caves and Subterranean Habitats (non-aquatic) - Caves7.2. Caves and Subterranean Habitats (non-aquatic) - Other Subterranean Habitats

#### Habitat

Habitat importance: Major Importance

Habitats: 7.1. Caves and Subterranean Habitats (non-aquatic) - Caves7.2. Caves and Subterranean Habitats (non-aquatic) - Other Subterranean Habitats

#### Ecology

Size: 4.85 mm (lectotype)

Generation length (yr): 1

Dependency of single sp?: Unknown

Ecology and traits (narrative): *Trechusmachadoi* is a troglobiont with reduced eyes and body depigmentation. It lives exclusively in subterranean habitats and is only known from a single cave and from scree slopes habitats in the Serra dos Candeeiros subunit of the Estremenho karst massif in central Portugal (Eusébio 2020, Reboleira 2007, Reboleira et al. 2009).

#### Threats

Justification for threats: Since the 1970s, this cave has been intensively explored for touristic activities. During that period, a second entrance has been opened, drastically changing the environment (Reboleira 2007, Reboleira et al. 2009). The scree slope, where it was recently found, is disturbed by herds of grazing goats (Eusébio 2020, Eusébio et al. 2021). Both sites are located 640 m from a field of energy windmills, 1 km from a quarry, 850 m from agricultural lands and 690 m from the nearest village.

##### Threats

Threat type: Ongoing

Threats: 1.1. Residential & commercial development - Housing & urban areas3.2. Energy production & mining - Mining & quarrying3.3. Energy production & mining - Renewable energy6.1. Human intrusions & disturbance - Recreational activities

#### Threats

Threat type: Ongoing

Threats: 1.1. Residential & commercial development - Housing & urban areas3.2. Energy production & mining - Mining & quarrying3.3. Energy production & mining - Renewable energy6.1. Human intrusions & disturbance - Recreational activities

#### Conservation

Justification for conservation actions: The habitats are protected under the EU “Rede Natura 2000” (Directive 1992, ICNB 2000), but the species lacks a formal protection framework. Monitoring of the population trends and extent of distribution in the underground in the nearby areas is crucial to understand the species' abundance patterns and life cycle. Improvements are needed to reduce the impacts of the quarry and windmills on the habitat.

##### Conservation actions

Conservation action type: Needed

Conservation actions: 1.1. Land/water protection - Site/area protection2.1. Land/water management - Site/area management4. Education & awareness5.1.3. Law & policy - Legislation - Sub-national level

#### Conservation actions

Conservation action type: Needed

Conservation actions: 1.1. Land/water protection - Site/area protection2.1. Land/water management - Site/area management4. Education & awareness5.1.3. Law & policy - Legislation - Sub-national level

#### Other

##### Use and trade

Use type: International

##### Ecosystem services

Ecosystem service type: Very important

##### Research needed

Research needed: 1.2. Research - Population size, distribution & trends1.3. Research - Life history & ecology2.2. Conservation Planning - Area-based Management Plan3.1. Monitoring - Population trends3.4. Monitoring - Habitat trends

Justification for research needed: Further investigation is needed about the population size, extent of distribution, ecology and life cycle. It is urgent to develop a management plan for this species, which will consequently improve the conservation of further cave-adapted species of Serra dos Candeeiros subunit of the Estremenho massif.

#### Use and trade

Use type: International

#### Ecosystem services

Ecosystem service type: Very important

#### Research needed

Research needed: 1.2. Research - Population size, distribution & trends1.3. Research - Life history & ecology2.2. Conservation Planning - Area-based Management Plan3.1. Monitoring - Population trends3.4. Monitoring - Habitat trends

Justification for research needed: Further investigation is needed about the population size, extent of distribution, ecology and life cycle. It is urgent to develop a management plan for this species, which will consequently improve the conservation of further cave-adapted species of Serra dos Candeeiros subunit of the Estremenho massif.

#### Viability analysis

### Trechus gamae

#### Species information

Scientific name: Trechusgamae

Species authority: Ribeira & Reboleira, 2009

Kingdom: Animalia

Phylum: Arthropoda

Class: Insecta

Order: Coleoptera

Family: Carabidae

Taxonomic notes: This species belongs to the “*T.fulvus*-group” species complex.

**Figure 3.**
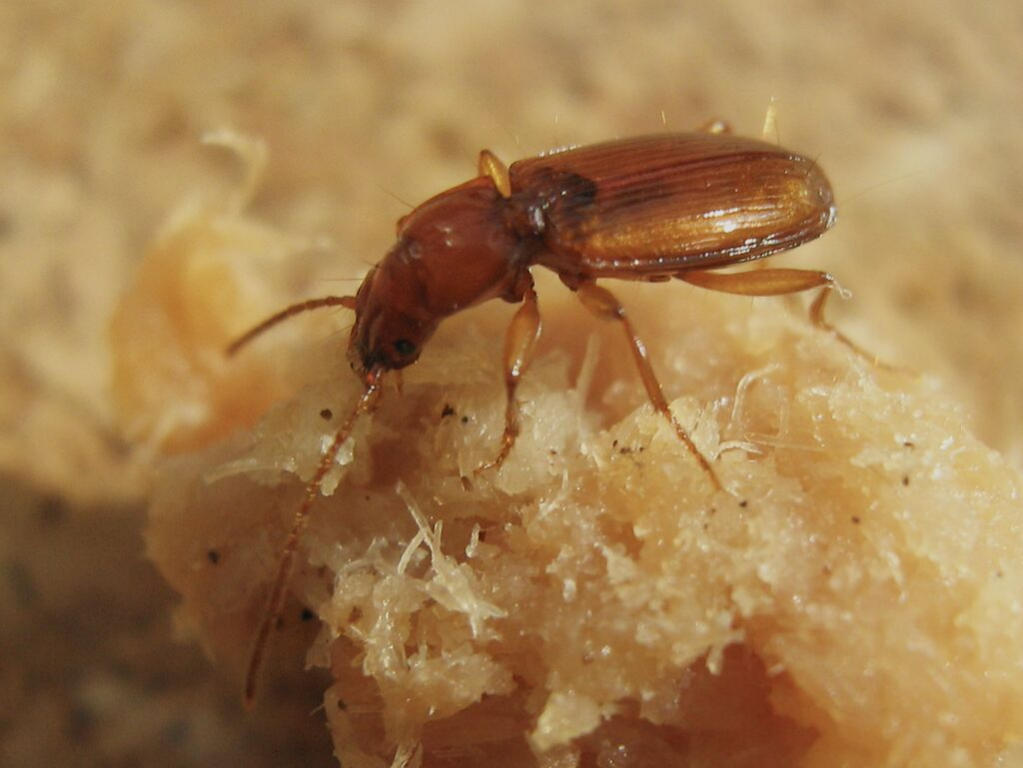
*Trechusgamae* Reboleira & Serrano, 2009. Algar do Pena Cave, Estremenho karst massif, Portugal.

Figure(s) or Photo(s): Fig. 3

Region for assessment: Europe

#### Geographic range

Biogeographic realm: Palearctic

Countries: Portugal

Map of records (Google Earth): Suppl. materials 1, 4

Basis of EOO and AOO: Known habitat extent

Basis (narrative): The extent of occurrence (EOO) is 73.4 km² and the maximum estimated area of occupancy (AOO) is 24 km².

Min Elevation/Depth (m): 250

Max Elevation/Depth (m): 485

Range description: *Trechusgamae* is a troglobiont beetle, known from five caves and from the mesovoid shallow substrate (scree slopes), all located in the Santo António Plateau, the central subunit of the Estremenho karst massif (Eusébio 2020, Eusébio et al. 2021, Reboleira 2007, Reboleira 2012, Reboleira and Ortuño 2011, Reboleira et al. 2009).

#### New occurrences

#### Extent of occurrence

EOO (km2): 73.4

Trend: Unknown

Causes ceased?: Unknown

Causes understood?: Unknown

Causes reversible?: Unknown

Extreme fluctuations?: Unknown

#### Area of occupancy

Trend: Unknown

Causes ceased?: Unknown

Causes understood?: Unknown

Causes reversible?: Unknown

Extreme fluctuations?: Unknown

AOO (km2): 24

#### Locations

Number of locations: 6

Justification for number of locations: *Trechusgamae* was found in five caves: Algar de Marradinhas II, Algar das Gralhas VII, Algar do Pena, Algar da Arroteia and Algar do Ladoeiro, all located in the Santo António Plateau, the central subunit of the Estremenho karst massif. Recently, a single specimen was found in the mesovoid shallow substrate at 0.5 m depth in scree slopes of Fórnea, which is also located in the Santo António Plateau, showing that this species may also disperse through more superficial subterranean habitats (Eusébio 2020). The species is geographically isolated within the subterranean network of the Estremenho's massif subunit Santo António Plateau (Eusébio 2020, Reboleira 2007, Reboleira 2012, Reboleira and Ortuño 2011, Reboleira et al. 2009, Reboleira et al. 2010).

Trend: Unknown

Extreme fluctuations?: Unknown

#### Population

Number of individuals: Unknown

Trend: Unknown

Causes ceased?: Unknown

Causes understood?: Unknown

Causes reversible?: Unknown

Extreme fluctuations?: Unknown

Population Information (Narrative): Amongst the six known localities with populations of *T.gamae*, Algar das Gralhas VII Cave had the largest population, followed by Algar do Pena Cave, Algar das Marradinhas Cave and Algar da Arroteia Cave (Reboleira 2007, Reboleira and Ortuño 2011). Algar do Ladoeiro Cave and the mesovoid shallow substrate in scree slopes in Fórnea (contiguous to Algar da Arroteia Cave) had the smallest population (Reboleira et al. 2009; Eusébio et al. 2021). All the known populations are within the Santo António Plateau.

#### Subpopulations

Number of subpopulations: 6

Trend: Decline (inferred)

Justification for trend: All the subpopulations face threats derived from intensive quarrying activity, which changes land use and disturbs the natural processes of the habitat. The subpopulations from Algar do Ladoeiro, Algar das Marradinhas II, Algar da Arroteia and Fórnea face threats of pollution and land use disturbance due to the proximity of urbanised areas.

Extreme fluctuations?: Unknown

Severe fragmentation?: No

#### Habitat

System: Terrestrial

Habitat specialist: Yes

Habitat (narrative): *Trechusgamae* was found in the deepest parts of the caves, from 50 to 95 m depth, all with high humidity levels (> 98%) and temperatures ranging from 13.5ºC (in Algar do Pena) to 17ºC (in Algar de Marradinhas II) (Reboleira 2007, Reboleira 2012, Reboleira and Ortuño 2011, Reboleira et al. 2009). The caves where the species were found are located at an altitude ranging from 250 to 485 m a.s.l. (Reboleira and Ortuño 2011). More recently, a single specimen was collected in MSS traps in scree slopes in Fórnea, which is located between the Algar da Arroteia and the Algar do Ladoeiro Caves. The MSS specimen was collected in the winter, in a pitfall trap placed at 50 cm deep into the scree slope (Eusébio et al. 2021).

Trend in extent, area or quality?: Decline (inferred)

##### Habitat

Habitat importance: Major Importance

Habitats: 7.1. Caves and Subterranean Habitats (non-aquatic) - Caves7.2. Caves and Subterranean Habitats (non-aquatic) - Other Subterranean Habitats

#### Habitat

Habitat importance: Major Importance

Habitats: 7.1. Caves and Subterranean Habitats (non-aquatic) - Caves7.2. Caves and Subterranean Habitats (non-aquatic) - Other Subterranean Habitats

#### Ecology

Size: 4.83–5.38 mm (males), 3.94–5.44 mm (females)

Generation length (yr): 1

Dependency of single sp?: Unknown

Ecology and traits (narrative): *Trechusgamae* was the only cave-adapted beetle collected in the caves and MSS of the Santo António Plateau and it shows a strict subterranean lifestyle (Eusébio et al. 2021, Reboleira 2007, Reboleira 2012, Reboleira and Ortuño 2011, Reboleira et al. 2009, Reboleira et al. 2010). Both adults and larvae were found in the sampled caves and some seasonal abundance variation was recorded, being more abundant in spring and summer (Reboleira and Ortuño 2011).

#### Threats

Justification for threats: The distribution area of the species is all covered by the Natural Park of Serras d'Aire e Candeeiros. However, intense quarry activity is currently ongoing in the surrounding areas of the known localities. Algar do Pena is located 300 m from a quarry and Algar das Gralhas VII 168 m from the same quarry. Algar do Ladoeiro's entrance is 840 m from the closest urban areas, Algar das Marradinhas II is located 1.5 km from the nearest village and Algar da Arroteia is located 112 m from the closest house and 1.3 km from a quarry. Algar do Pena Cave hosts a laboratory and is open for visits upon previous booking. Fórnea is located 595 m from the closest house and 1.9 km from a quarry. The distribution area faces severe groundwater contamination (Reboleira et al. 2013b), due to the generalised lack of proper wastewater treatment (Reboleira et al. 2011b).

##### Threats

Threat type: Ongoing

Threats: 1.1. Residential & commercial development - Housing & urban areas3.2. Energy production & mining - Mining & quarrying6.1. Human intrusions & disturbance - Recreational activities9.1.2. Pollution - Domestic & urban waste water - Run-off

#### Threats

Threat type: Ongoing

Threats: 1.1. Residential & commercial development - Housing & urban areas3.2. Energy production & mining - Mining & quarrying6.1. Human intrusions & disturbance - Recreational activities9.1.2. Pollution - Domestic & urban waste water - Run-off

#### Conservation

Justification for conservation actions: Although the habitat is protected by law under the “Rede Natura 2000” (Directive 1992, ICNB 2000), the species is not. Population trends need to be monitored in order to better understand the species' abundance patterns and life cycle. Measures should be taken to prevent infiltration of wastewaters from the nearby town into the soil and underground habitats and to minimise the negative effects of the quarry in the habitat.

##### Conservation actions

Conservation action type: Needed

Conservation actions: 1.1. Land/water protection - Site/area protection2.1. Land/water management - Site/area management4. Education & awareness5.1.3. Law & policy - Legislation - Sub-national level

#### Conservation actions

Conservation action type: Needed

Conservation actions: 1.1. Land/water protection - Site/area protection2.1. Land/water management - Site/area management4. Education & awareness5.1.3. Law & policy - Legislation - Sub-national level

#### Other

##### Use and trade

Use type: International

##### Ecosystem services

Ecosystem service type: Very important

##### Research needed

Research needed: 1.2. Research - Population size, distribution & trends1.3. Research - Life history & ecology2.2. Conservation Planning - Area-based Management Plan3.1. Monitoring - Population trends3.4. Monitoring - Habitat trends

Justification for research needed: Additional investigation about the population size, extent of distribution, ecology and life cycle is required. The development of a management plan that will improve the conservation of this cave-adapted species in the Santo António Plateau subunit of the Estremenho karst massif is fundamental to ensure its conservation and protection.

#### Use and trade

Use type: International

#### Ecosystem services

Ecosystem service type: Very important

#### Research needed

Research needed: 1.2. Research - Population size, distribution & trends1.3. Research - Life history & ecology2.2. Conservation Planning - Area-based Management Plan3.1. Monitoring - Population trends3.4. Monitoring - Habitat trends

Justification for research needed: Additional investigation about the population size, extent of distribution, ecology and life cycle is required. The development of a management plan that will improve the conservation of this cave-adapted species in the Santo António Plateau subunit of the Estremenho karst massif is fundamental to ensure its conservation and protection.

#### Viability analysis

### Trechus lunai

#### Species information

Scientific name: Trechuslunai

Species authority: Ribeira & Reboleira, 2009

Kingdom: Animalia

Phylum: Arthropoda

Class: Insecta

Order: Coleoptera

Family: Carabidae

Taxonomic notes: This species belongs to the “*T.fulvus*-group” species complex.

**Figure 4.**
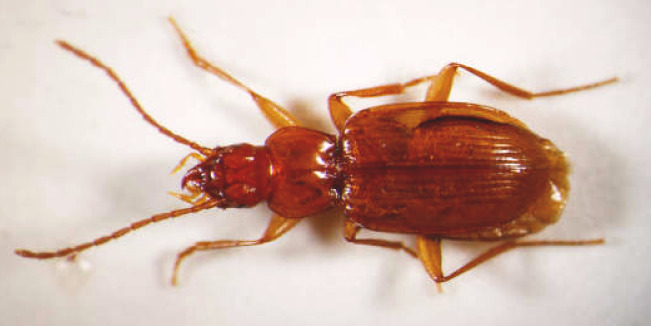
*Trechuslunai* Reboleira & Serrano, 2009. Almonda Cave, Estremenho karst massif, Portugal.

Figure(s) or Photo(s): Fig. 4

Region for assessment: Europe

#### Geographic range

Biogeographic realm: Palearctic

Countries: Portugal

Map of records (Google Earth): Suppl. materials 1, 5

Basis of EOO and AOO: Known habitat extent

Basis (narrative): The extent of occurrence (EOO) is 4 km^2^ and the maximum estimated area of occupancy (AOO) is 12 km².

Min Elevation/Depth (m): 95

Max Elevation/Depth (m): 307

Range description: *Trechuslunai* is a troglobiont Carabidae known from three horizontal caves, located in Serra de Aire/São Mamede Plateau (Reboleira et al. 2009).

#### New occurrences

#### Extent of occurrence

EOO (km2): 4

Trend: Unknown

Causes ceased?: Unknown

Causes understood?: Unknown

Causes reversible?: Unknown

Extreme fluctuations?: Unknown

#### Area of occupancy

Trend: Unknown

Causes ceased?: Unknown

Causes understood?: Unknown

Causes reversible?: Unknown

Extreme fluctuations?: Unknown

AOO (km2): 12

#### Locations

Number of locations: 3

Justification for number of locations: *Trechuslunai* was found in three caves of the Serra de Aire/São Mamede Plateau subunits. The southernmost distribution is the Almonda Cave, but this species is also known from the Contenda and Moinhos Velhos Caves' system (Reboleira et al. 2009).

Trend: Unknown

Extreme fluctuations?: Unknown

#### Population

Number of individuals: Unknown

Trend: Unknown

Causes ceased?: Unknown

Causes understood?: Unknown

Causes reversible?: Unknown

Extreme fluctuations?: Unknown

Population Information (Narrative): Three populations are known exclusively from the Serra d'Aire/São Mamede Plateau subunit of the Estremenho karst massif (Reboleira et al. 2009).

#### Subpopulations

Number of subpopulations: 3

Trend: Decline (inferred)

Justification for trend: The subpopulation in Almonda cave is subject to wastewater and pollution infiltration and the subpopulations of the Contenda and Moinhos Velhos Caves face heavy contamination derived from the village under which they are located.

Extreme fluctuations?: Unknown

Severe fragmentation?: No

#### Habitat

System: Terrestrial

Habitat specialist: Yes

Habitat (narrative): *Trechuslunai* only occurs in the deepest parts of the caves, from 50 to 80 m depth. The three caves have high humidity levels and average temperature of 18ºC (Reboleira et al. 2009, Reboleira et al. 2015). In Moinhos Velhos Cave, the species was found in a subterranean stream that has high input of sewage from surface and is located below the village of Mira d'Aire in the connection between the Gruta de Mira d'Aire Show Cave and the Pena Spring. Moinhos Velhos is contiguous to Contenda Cave and are hydrologically connected (Reboleira et al. 2009). The other locality is Almonda Cave, which is located 8.4 km in a straight line from the Moinhos Velhos-Contenda cave system. Almonda Cave, also known as “Olho do Moinho da Fonte”, is the largest cave of Portugal with more than 10 km of horizontal development mapped (Thomas 1991).

Trend in extent, area or quality?: Decline (inferred)

##### Habitat

Habitat importance: Major Importance

Habitats: 7.1. Caves and Subterranean Habitats (non-aquatic) - Caves

#### Habitat

Habitat importance: Major Importance

Habitats: 7.1. Caves and Subterranean Habitats (non-aquatic) - Caves

#### Ecology

Size: 3.55–4.73 mm

Generation length (yr): 1

Dependency of single sp?: Unknown

Ecology and traits (narrative): *Trechuslunai* was the only troglobiont species captured in these caves. All known localities are caves that flood seasonally (Reboleira et al. 2009).

#### Threats

Justification for threats: Almonda Cave is located 50 m from a factory that conducts the subterranean river into the building for industrial use of the water and at 420 m from the village centre. The surrounding village is also heavily populated by agricultural fields. Contenda and Moinhos Velhos caves are heavily contaminated, as their subterranean network extends below the village of Mira d'Aire. The entrance of Moinhos Velhos Cave is located in the village centre and it has been explored for tourism since the 1960s with several complementary touristic infrastructures built (Crispim et al. 2014).

##### Threats

Threat type: Ongoing

Threats: 1.1. Residential & commercial development - Housing & urban areas1.2. Residential & commercial development - Commercial & industrial areas2.3. Agriculture & aquaculture - Livestock farming & ranching9.1.2. Pollution - Domestic & urban waste water - Run-off

#### Threats

Threat type: Ongoing

Threats: 1.1. Residential & commercial development - Housing & urban areas1.2. Residential & commercial development - Commercial & industrial areas2.3. Agriculture & aquaculture - Livestock farming & ranching9.1.2. Pollution - Domestic & urban waste water - Run-off

#### Conservation

Justification for conservation actions: The Contenda and Moinhos-Velhos caves develop below the village of Mira d'Aire and infiltration of sewage is observed in the underground. Therefore an effort to improve sewage treatment is necessary in order to prevent wastewater run-off into subterranean gealleries and groundwaters. Almonda Cave is classified as Property of Public Interest (IIP) since 1993 and protected due to archaeological heritage (Hoffmann et al. 2013). The archaeological arguments for cave protection are clearly inappropriate for cave-adapted fauna conservation, so it is urgent to set specific measures to ensure its protection.

##### Conservation actions

Conservation action type: Needed

Conservation actions: 1.1. Land/water protection - Site/area protection2.1. Land/water management - Site/area management4. Education & awareness5.1.3. Law & policy - Legislation - Sub-national level

#### Conservation actions

Conservation action type: Needed

Conservation actions: 1.1. Land/water protection - Site/area protection2.1. Land/water management - Site/area management4. Education & awareness5.1.3. Law & policy - Legislation - Sub-national level

#### Other

##### Use and trade

Use type: International

##### Ecosystem services

Ecosystem service type: Very important

##### Research needed

Research needed: 1.2. Research - Population size, distribution & trends1.3. Research - Life history & ecology2.2. Conservation Planning - Area-based Management Plan3.1. Monitoring - Population trends3.4. Monitoring - Habitat trends

Justification for research needed: Information about the population size, extent of distribution, ecology and life cycle of this species is scarce, therefore, further investigation is required. It is also necessary to develop a management plan that will improve the conservation of the species in the Serra de Aire/S. Mamede Plateau subunits of the Estremenho karst massif.

#### Use and trade

Use type: International

#### Ecosystem services

Ecosystem service type: Very important

#### Research needed

Research needed: 1.2. Research - Population size, distribution & trends1.3. Research - Life history & ecology2.2. Conservation Planning - Area-based Management Plan3.1. Monitoring - Population trends3.4. Monitoring - Habitat trends

Justification for research needed: Information about the population size, extent of distribution, ecology and life cycle of this species is scarce, therefore, further investigation is required. It is also necessary to develop a management plan that will improve the conservation of the species in the Serra de Aire/S. Mamede Plateau subunits of the Estremenho karst massif.

#### Viability analysis

### Trechus tatai

#### Species information

Scientific name: Trechustatai

Species authority: Reboleira & Ortuño, 2010

Kingdom: Animalia

Phylum: Arthropoda

Class: Insecta

Order: Coleoptera

Family: Carabidae

Taxonomic notes: This species belongs to the “*T.fulvus*-group” species complex. It is recognisable by the shape of the aedeagus and has a slim body, rudimentary wings, reduced eyes (microphthalmia) and depigmentation. This species is the most troglomorphic ground-beetle known from Portugal (Reboleira et al. 2010).

**Figure 5.**
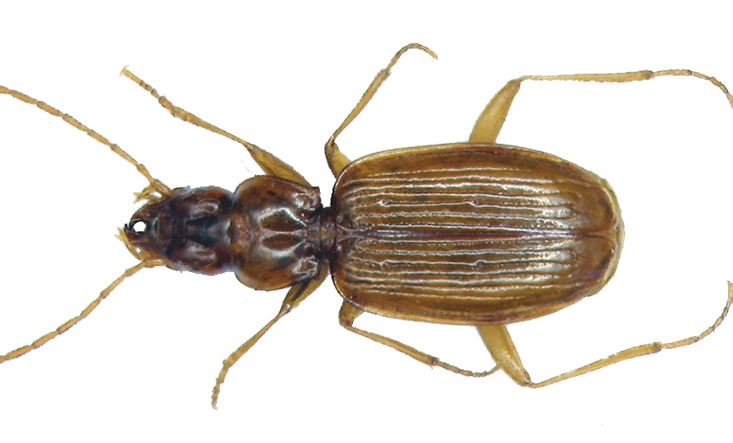
*Trechustatai* Reboleira & Ortuño, 2010, Algar do Javali Cave, Montejunto karst massif, Portugal.

Figure(s) or Photo(s): Fig. 5

Region for assessment: Europe

#### Geographic range

Biogeographic realm: Palearctic

Countries: Portugal

Map of records (Google Earth): Suppl. materials 1, 6

Basis of EOO and AOO: Known habitat extent

Basis (narrative): The extent of occurrence (EOO) and the maximum estimated area of occupancy (AOO) are both of 4 km².

Min Elevation/Depth (m): 380

Range description: *Trechustatai* is a cave-adapted hygrophilous Carabidae known only from one small cave, located in Serra do Montejunto (Reboleira et al. 2010).

#### New occurrences

#### Extent of occurrence

EOO (km2): 4

Trend: Unknown

Causes ceased?: Unknown

Causes understood?: Unknown

Causes reversible?: Unknown

Extreme fluctuations?: Unknown

#### Area of occupancy

Trend: Unknown

Causes ceased?: Unknown

Causes understood?: Unknown

Causes reversible?: Unknown

Extreme fluctuations?: Unknown

AOO (km2): 4

#### Locations

Number of locations: 1

Justification for number of locations: *Trechustatai* only occurs in one cave, Algar do Javali, located in Serra do Montejunto. This species is geographically isolated (Reboleira et al. 2010).

Trend: Stable

Justification for trend: Algar do Javali is the only known location for this species, therefore, the trend in number of locations is stable.

Extreme fluctuations?: Unknown

#### Population

Number of individuals: Unknown

Trend: Unknown

Causes ceased?: Unknown

Causes understood?: Unknown

Causes reversible?: Unknown

Extreme fluctuations?: Unknown

Population Information (Narrative): Only one population is known from Algar do Javali Cave, in Montejunto karst massif.

#### Subpopulations

Trend: Unknown

Extreme fluctuations?: Unknown

Severe fragmentation?: No

#### Habitat

System: Terrestrial

Habitat specialist: Yes

Habitat (narrative): *Trechustatai* was only collected in the deep oligotrophic areas of the cave. It was never found in areas with high organic material content (bat guano accumulation zones). The cave is 10 m deep and extends for 80 m. Temperatures in the deepest zone of the cave ranged from 14.2 ºC in winter to 15 ºC in summer (Reboleira et al. 2010).

Trend in extent, area or quality?: Decline (inferred)

##### Habitat

Habitat importance: Major Importance

Habitats: 7.1. Caves and Subterranean Habitats (non-aquatic) - Caves

#### Habitat

Habitat importance: Major Importance

Habitats: 7.1. Caves and Subterranean Habitats (non-aquatic) - Caves

#### Ecology

Size: 5.2–5.9 mm (males), 4.8–6 mm (females)

Generation length (yr): 1

Dependency of single sp?: Unknown

Ecology and traits (narrative): *Trechustatai* is the most troglomorphic carabid beetle from continental Portugal. Other caves of the area were sampled, but *T.tatai* was never collected elsewhere. The seasonal activity pattern of the beetle was studied during one year in the deepest zone of the cave and specimens were collected during winter, autumn and spring (Reboleira et al. 2010). The absence of *T.tatai* during summer is most likely caused by the low humidity in the cave during this season. It is very likely that, during summer, this species escapes from the cave into the MSS in search of smaller gaps that retain humidity (Reboleira et al. 2010).

#### Threats

Justification for threats: Algar do Javali Cave is located 1.6 km from a quarry with intensive extraction activity and 2.9 km from the closest village, which induces deep changes in land use at the surface and potential biotic exchange, such as introduction of invasive alien species. The cave entrance is located 50 m from a road and surrounded by *Eucalyptus* intensive plantation, with direct impact on land use at the surface, pollution and groundwater depletion.

##### Threats

Threat type: Ongoing

Threats: 1.1. Residential & commercial development - Housing & urban areas2.2. Agriculture & aquaculture - Wood & pulp plantations3.2. Energy production & mining - Mining & quarrying4. Transportation & service corridors

#### Threats

Threat type: Ongoing

Threats: 1.1. Residential & commercial development - Housing & urban areas2.2. Agriculture & aquaculture - Wood & pulp plantations3.2. Energy production & mining - Mining & quarrying4. Transportation & service corridors

#### Conservation

Justification for conservation actions: Although this cave is protected by law through the “Rede Natura 2000” (Directive 1992, ICNB 2000), the species is not. This species is rare, a single cave endemic and it is considered the most troglomorphic carabid beetle of Portugal; therefore, a conservation plan for this cave area is crucial to ensure its environmental sustainability.

##### Conservation actions

Conservation action type: Needed

Conservation actions: 1.1. Land/water protection - Site/area protection2.1. Land/water management - Site/area management4. Education & awareness5.1.3. Law & policy - Legislation - Sub-national level

#### Conservation actions

Conservation action type: Needed

Conservation actions: 1.1. Land/water protection - Site/area protection2.1. Land/water management - Site/area management4. Education & awareness5.1.3. Law & policy - Legislation - Sub-national level

#### Other

##### Use and trade

Use type: International

##### Ecosystem services

Ecosystem service type: Very important

##### Research needed

Research needed: 1.2. Research - Population size, distribution & trends1.3. Research - Life history & ecology2.2. Conservation Planning - Area-based Management Plan3.1. Monitoring - Population trends3.4. Monitoring - Habitat trends

Justification for research needed: Some crucial steps necessary for the protection of the species are the development of a management plan for the conservation of this cave-adapted species in Serra do Montejunto and the promotion of further studies regarding population size, extent of distribution, ecology and life cycle.

#### Use and trade

Use type: International

#### Ecosystem services

Ecosystem service type: Very important

#### Research needed

Research needed: 1.2. Research - Population size, distribution & trends1.3. Research - Life history & ecology2.2. Conservation Planning - Area-based Management Plan3.1. Monitoring - Population trends3.4. Monitoring - Habitat trends

Justification for research needed: Some crucial steps necessary for the protection of the species are the development of a management plan for the conservation of this cave-adapted species in Serra do Montejunto and the promotion of further studies regarding population size, extent of distribution, ecology and life cycle.

#### Viability analysis

### Speonemadus algarvensis

#### Species information

Scientific name: Speonemadusalgarvensis

Species authority: Reboleira, Fresneda & Salgado, 2017

Kingdom: Animalia

Phylum: Arthropoda

Class: Insecta

Order: Coleoptera

Family: Leiodidae

Taxonomic notes: This species is part of the "*Speonemadusescalerai*-group" and is recognisable by the equal/subequal length of the 2^nd^, 4^th^, 5^th^ and 7^th^ antennomeres and a slightly transverse and hexagonal pronotum (Reboleira et al. 2017).

Region for assessment: Europe

#### Geographic range

Biogeographic realm: Palearctic

Countries: Portugal

Map of records (Google Earth): Suppl. materials 1, 7

Basis of EOO and AOO: Known habitat extent

Basis (narrative): The extent of occurrence (EOO) is 7.4 km^2^ and the maximum estimated area of occupancy (AOO) is 12 km^2^. The three caves are located along a 48 km straight line, with Algarão do Remexido Cave being 23 km from Vale Telheiro and Vale Telheiro Cave being 25 km from Senhora Cave.

Min Elevation/Depth (m): 72

Max Elevation/Depth (m): 269

Range description: *Speonemadusalgarvensis* was collected in three caves in the southermost province of Portugal in the Algarve, being most likely endemic to the central and eastern parts of the Algarve karst massif, a region also known as Barrocal Algarvio (Reboleira et al. 2017).

#### New occurrences

#### Extent of occurrence

EOO (km2): 7.4

Trend: Unknown

Causes ceased?: Unknown

Causes understood?: Unknown

Causes reversible?: Unknown

Extreme fluctuations?: Unknown

#### Area of occupancy

Trend: Unknown

Causes ceased?: Unknown

Causes understood?: Unknown

Causes reversible?: Unknown

Extreme fluctuations?: Unknown

AOO (km2): 12

#### Locations

Number of locations: 3

Justification for number of locations: *Speonemadusalgarvensis* is known from three caves (Vale Telheiro, Algarão do Remexido and Senhora) in the Algarve karst massif (Reboleira et al. 2017).

Trend: Unknown

Extreme fluctuations?: Unknown

#### Population

Number of individuals: Unknown

Trend: Unknown

Causes ceased?: Unknown

Causes understood?: Unknown

Causes reversible?: Unknown

Extreme fluctuations?: Unknown

Population Information (Narrative): There are three populations known from Portugal, all from caves in the Algarve karst massif. The largest number of individuals was collected in Vale Telheiro Cave, followed by Senhora Cave and Algarão do Remexido Cave (Reboleira et al. 2017).

#### Subpopulations

Number of subpopulations: 3

Trend: Decline (inferred)

Justification for trend: All populations are under risk due to wastewater infiltration derived from the urbanised areas in the region. The subpopulation of Algarão do Remexido cave is threatened by agricultural pollution infiltration and the subpopulation from Senhora cave is threatened by industrial residue pollution.

Extreme fluctuations?: Unknown

Severe fragmentation?: Unknown

#### Habitat

System: Terrestrial

Habitat specialist: Yes

Habitat (narrative): Specimens were collected in three caves, 15 to 30 m deep, with high humidity levels and average temperatures of 17.8ºC (Vale Telheiro Cave), 18.8ºC (Senhora Cave) and 19.3ºC (Algarão do Remexido Cave). This species is endemic to the Algarve karst massif (Reboleira et al. 2017).

Trend in extent, area or quality?: Decline (inferred)

##### Habitat

Habitat importance: Major Importance

Habitats: 7.1. Caves and Subterranean Habitats (non-aquatic) - Caves

#### Habitat

Habitat importance: Major Importance

Habitats: 7.1. Caves and Subterranean Habitats (non-aquatic) - Caves

#### Ecology

Size: 4–4.9 mm

Generation length (yr): 1

Dependency of single sp?: Unknown

Ecology and traits (narrative): *Speonemadusalgarvensis* occurs exclusively in the Algarve and does not exhibit the typical troglomorphism found in other cave-adapted species, such as evident eye reduction, severe depigmentation and extreme body and appendages elongation, although it has only been collected in caves and never at the surface. This species was found to carry the ectoparasitic fungus of the order Laboulbeniales (*Stichomycesconosomatis* Thaxt., 1901) attached to the cuticle and foresic acari (Reboleira et al. 2017).

#### Threats

Justification for threats: Algarão do Remexido is located under agricultural lands, 370 m from the closest house and 1.7 km from the closest village. Vale Telheiro is located 290 m from the closest house and 745 m from the closest urbanisation. Senhora Cave is located 168 m from the closest house and 900 m from an industrial complex.

##### Threats

Threat type: Ongoing

Threats: 1.1. Residential & commercial development - Housing & urban areas1.2. Residential & commercial development - Commercial & industrial areas2.3. Agriculture & aquaculture - Livestock farming & ranching9.1.2. Pollution - Domestic & urban waste water - Run-off

#### Threats

Threat type: Ongoing

Threats: 1.1. Residential & commercial development - Housing & urban areas1.2. Residential & commercial development - Commercial & industrial areas2.3. Agriculture & aquaculture - Livestock farming & ranching9.1.2. Pollution - Domestic & urban waste water - Run-off

#### Conservation

Justification for conservation actions: Although the habitat is protected under legislation by the “Rede Natura 2000” (Directive 1992, ICNB 2000), the species is not. Population trends need to be monitored in order to better understand the species' abundance patterns and life cycle. Measures should be taken to prevent infiltration of wastewaters and to ensure conservation of the natural landscape and plant communities on the surface, which are necessary to maintain nutrient inflow to the subterranean ecosystem.

##### Conservation actions

Conservation action type: Needed

Conservation actions: 1.1. Land/water protection - Site/area protection2.1. Land/water management - Site/area management4. Education & awareness5.1.3. Law & policy - Legislation - Sub-national level

#### Conservation actions

Conservation action type: Needed

Conservation actions: 1.1. Land/water protection - Site/area protection2.1. Land/water management - Site/area management4. Education & awareness5.1.3. Law & policy - Legislation - Sub-national level

#### Other

##### Use and trade

Use type: International

##### Ecosystem services

Ecosystem service type: Very important

##### Research needed

Research needed: 1.2. Research - Population size, distribution & trends1.3. Research - Life history & ecology2.2. Conservation Planning - Area-based Management Plan3.1. Monitoring - Population trends3.4. Monitoring - Habitat trends

Justification for research needed: The development of a management plan for the conservation of this cave-adapted species in the Algarve karst massif and the encouragement of more studies regarding population size, extent of distribution, ecology and life cycle are essential measures for the protection of the species.

#### Use and trade

Use type: International

#### Ecosystem services

Ecosystem service type: Very important

#### Research needed

Research needed: 1.2. Research - Population size, distribution & trends1.3. Research - Life history & ecology2.2. Conservation Planning - Area-based Management Plan3.1. Monitoring - Population trends3.4. Monitoring - Habitat trends

Justification for research needed: The development of a management plan for the conservation of this cave-adapted species in the Algarve karst massif and the encouragement of more studies regarding population size, extent of distribution, ecology and life cycle are essential measures for the protection of the species.

#### Viability analysis

### Domene lusitanica

#### Species information

Scientific name: Domenelusitanica

Species authority: Reboleira & Oromí, 2011

Kingdom: Animalia

Phylum: Arthropoda

Class: Insecta

Order: Coleoptera

Family: Staphylinidae

Taxonomic notes: Individuals display troglomorphism, such as microphthalmia, lack of wings and body elongation (Reboleira et al. 2011a).

**Figure 6.**
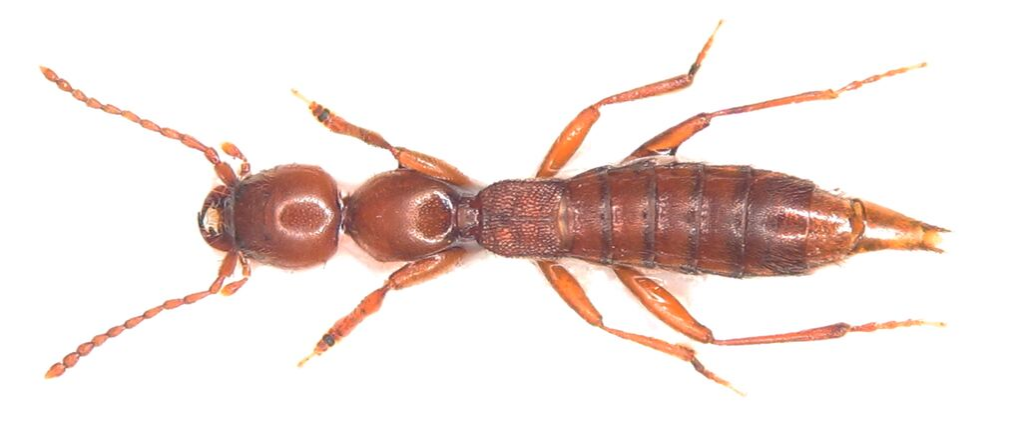
*Domenelusitanica* Reboleira & Oromí, 2011, from Cerâmica Cave in Sicó karst area, central Portugal.

Figure(s) or Photo(s): Fig. 6

Region for assessment: Europe

#### Geographic range

Biogeographic realm: Palearctic

Countries: Portugal

Map of records (Google Earth): Suppl. materials 1, 8

Basis of EOO and AOO: Known habitat extent

Basis (narrative): The extent of occurrence (EOO) and the maximum estimated area of occupancy (AOO) are both of 4 km².

Range description: *Domenelusitanica* was found in a single cave located in the Sicó karstic massif (Reboleira et al. 2011a).

#### New occurrences

#### Extent of occurrence

EOO (km2): 4

Trend: Unknown

Causes ceased?: Unknown

Causes understood?: Unknown

Causes reversible?: Unknown

Extreme fluctuations?: Unknown

#### Area of occupancy

Trend: Unknown

Causes ceased?: Unknown

Causes understood?: Unknown

Causes reversible?: Unknown

Extreme fluctuations?: Unknown

AOO (km2): 4

#### Locations

Number of locations: 1

Justification for number of locations: *Domenelusitanica* was collected in Cerâmica Cave, located in the Sicó karst area in central Portugal (Reboleira et al. 2011a). This Ccave extends for 355 m (Nóbrega et al. 1984).

Trend: Unknown

Extreme fluctuations?: Unknown

#### Population

Number of individuals: Unknown

Trend: Unknown

Causes ceased?: Unknown

Causes understood?: Unknown

Causes reversible?: Unknown

Extreme fluctuations?: Unknown

Population Information (Narrative): This species is known from a single population in central Portugal (Reboleira et al. 2011a).

#### Subpopulations

Trend: Unknown

Extreme fluctuations?: Unknown

Severe fragmentation?: Unknown

#### Habitat

System: Terrestrial

Habitat specialist: Yes

Habitat (narrative): Specimens were exclusively collected in the deepest zones of the cave (10 m deep), in high humidity levels and with average temperatures of 16.4ºC (Reboleira et al. 2011a).

Trend in extent, area or quality?: Decline (inferred)

##### Habitat

Habitat importance: Major Importance

Habitats: 7.1. Caves and Subterranean Habitats (non-aquatic) - Caves

#### Habitat

Habitat importance: Major Importance

Habitats: 7.1. Caves and Subterranean Habitats (non-aquatic) - Caves

#### Ecology

Size: 9–9.48 mm

Generation length (yr): 1

Dependency of single sp?: Unknown

Ecology and traits (narrative): *Domenelusitanica* is included in the subgenus Lathromene, together with the other two Portuguese species of cave-adapted *Domene*: *D.viriatoi* and *D.darinkae*. This species is a predator troglobiont rove beetle, with reduced eyes, apterous, depigmented and elongated body and appendages (Reboleira et al. 2011a). It is only known from one cave in the Sicó karst area and is a rare species, as the type locality and several other caves nearby have been monitored for more than a decade and only eight specimens have been observed so far: seven collected in 2010 (type material) and one collected in December 2019. It shares habitat with other single cave endemic species, the troglobiont pseudoscopion *Roncocreagrisborgesi* Zaragoza & Reboleira, 2013 and several other cave-adapted species: the pseudoscorpions *Occidenchthoniusvachoni* Zaragoza & Reboleira, 2018 and *Roncocreagrisblothroides* Beier, 1962; the millipede *Scutogonaminor* Enghoff & Reboleira, 2013; and the woodlice *Trichoniscoidessicoensis* Reboleira & Taiti, 2015, *Miktoniscuslongispina* Reboleira & Taiti, 2015 and *Porcelliocavernicolus* Vandel, 1946 (Enghoff and Reboleira 2013, Reboleira et al. 2013c, Reboleira et al. 2015).

#### Threats

Justification for threats: Cerâmica Cave is located 550 m from an animal farm, 3.5 km from the nearest village and 3.6 km from a quarry. It is surrounded by agricultural lands and *Eucalyptus* plantations.

##### Threats

Threat type: Ongoing

Threats: 1.1. Residential & commercial development - Housing & urban areas2.3. Agriculture & aquaculture - Livestock farming & ranching3.2. Energy production & mining - Mining & quarrying

#### Threats

Threat type: Ongoing

Threats: 1.1. Residential & commercial development - Housing & urban areas2.3. Agriculture & aquaculture - Livestock farming & ranching3.2. Energy production & mining - Mining & quarrying

#### Conservation

Justification for conservation actions: The habitat is located in an “Rede Natura 2000” area (Directive 1992, ICNB 2000). Population trends need to be monitored in order to better understand the species' abundance patterns and life cycle. Measures should be taken to prevent infiltrations from agricultural lands and livestock farms and to prevent the pernicious effects of the quarry activity on the surrounding habitats.

##### Conservation actions

Conservation action type: Needed

Conservation actions: 1.1. Land/water protection - Site/area protection2.1. Land/water management - Site/area management4. Education & awareness5.1.3. Law & policy - Legislation - Sub-national level

#### Conservation actions

Conservation action type: Needed

Conservation actions: 1.1. Land/water protection - Site/area protection2.1. Land/water management - Site/area management4. Education & awareness5.1.3. Law & policy - Legislation - Sub-national level

#### Other

##### Use and trade

Use type: International

##### Ecosystem services

Ecosystem service type: Very important

##### Research needed

Research needed: 1.2. Research - Population size, distribution & trends1.3. Research - Life history & ecology2.2. Conservation Planning - Area-based Management Plan3.1. Monitoring - Population trends3.4. Monitoring - Habitat trends

Justification for research needed: In order to build a sustainable conservation plan for the species in the Sicó karst area, more information about population size, extent of distribution, ecology and life cycle is needed. The threats also need to be addressed and minimised, if possible, in order to improve the habitat quality.

#### Use and trade

Use type: International

#### Ecosystem services

Ecosystem service type: Very important

#### Research needed

Research needed: 1.2. Research - Population size, distribution & trends1.3. Research - Life history & ecology2.2. Conservation Planning - Area-based Management Plan3.1. Monitoring - Population trends3.4. Monitoring - Habitat trends

Justification for research needed: In order to build a sustainable conservation plan for the species in the Sicó karst area, more information about population size, extent of distribution, ecology and life cycle is needed. The threats also need to be addressed and minimised, if possible, in order to improve the habitat quality.

#### Viability analysis

### Domene viriatoi

#### Species information

Scientific name: Domeneviriatoi

Species authority: Serrano & Boieiro, 2015

Kingdom: Animalia

Phylum: Arthropoda

Class: Insecta

Order: Coleoptera

Family: Staphylinidae

Taxonomic notes: This species displays body, leg and antennae elongation, microphthalmia and lack of wings (Serrano et al. 2015).

Region for assessment: Europe

#### Geographic range

Biogeographic realm: Palearctic

Countries: Portugal

Map of records (Google Earth): Suppl. materials 1, 9

Basis of EOO and AOO: Known habitat extent

Basis (narrative): The extent of occurrence (EOO) and the maximum estimated area of occupancy (AOO) are both 4 km^2^.

Range description: *Domeneviriatoi* was collected in two galleries of the Buraco da Moura Cave, located in the Serra da Estrela Mountain foothills (Serrano et al. 2015).

#### New occurrences

#### Extent of occurrence

EOO (km2): 4

Trend: Unknown

Causes ceased?: Unknown

Causes understood?: Unknown

Causes reversible?: Unknown

Extreme fluctuations?: Unknown

#### Area of occupancy

Trend: Unknown

Causes ceased?: Unknown

Causes understood?: Unknown

Causes reversible?: Unknown

Extreme fluctuations?: Unknown

AOO (km2): 4

#### Locations

Number of locations: 1

Justification for number of locations: *Domeneviriatoi* was collected from the Buraco da Moura Cave at the edge of the Estrela Mountain chain (Serrano et al. 2015).

Trend: Unknown

Extreme fluctuations?: Unknown

#### Population

Number of individuals: Unknown

Trend: Unknown

Causes ceased?: Unknown

Causes understood?: Unknown

Causes reversible?: Unknown

Extreme fluctuations?: Unknown

Population Information (Narrative): This species is known from a single population in the western border of the Estrela Mountain chain, the highest mountain of continental Portugal (Serrano et al. 2015).

#### Subpopulations

Trend: Unknown

Extreme fluctuations?: Unknown

Severe fragmentation?: Unknown

#### Habitat

System: Terrestrial

Habitat specialist: Yes

Habitat (narrative): The cave is formed by granite blocks in the margins of the Caniça stream at an elevation of 677 m. It extends for 150 m of underground passages (Serrano et al. 2015).

Trend in extent, area or quality?: Decline (inferred)

##### Habitat

Habitat importance: Major Importance

Habitats: 7.1. Caves and Subterranean Habitats (non-aquatic) - Caves

#### Habitat

Habitat importance: Major Importance

Habitats: 7.1. Caves and Subterranean Habitats (non-aquatic) - Caves

#### Ecology

Size: 6.9‒8.2 mm (males), 6.3‒8.8 mm (females)

Generation length (yr): 1

Dependency of single sp?: Unknown

Ecology and traits (narrative): *Domeneviriatoi* is included in the subgenus Lathromene. Both adults and larvae of this species were observed foraging for preys in bat guano on the cave substrate (Serrano et al. 2015). The cave is formed by blocks of granite and has considerable humidity. This species shares habitat with other cave species, like the millipede *Lusitanipusalternans* and the dipluran Podocampacf.fragiloides.

#### Threats

Justification for threats: The cave entrance is located 127 m from the closest house, 530 m from a hydroelectric power station and 1.2 km from the closest village and is under anthropogenic disturbance due to tourism.

##### Threats

Threat type: Ongoing

Threats: 1.1. Residential & commercial development - Housing & urban areas6.1. Human intrusions & disturbance - Recreational activities7.2. Natural system modifications - Dams & water management/use9.1.2. Pollution - Domestic & urban waste water - Run-off

#### Threats

Threat type: Ongoing

Threats: 1.1. Residential & commercial development - Housing & urban areas6.1. Human intrusions & disturbance - Recreational activities7.2. Natural system modifications - Dams & water management/use9.1.2. Pollution - Domestic & urban waste water - Run-off

#### Conservation

Justification for conservation actions: Buraco da Moura Cave was classified as a “National Important Underground Shelter for Bats” therefore a decrease in human disturbance is expected (Serrano et al. 2015). However, specific monitoring plans for this species are required in order to understand its ecology and distribution.Although the habitat is protected under legislation by the “Rede Natura 2000” (Directive 1992, ICNB 2000), the species is not. Population trends need to be monitored in order to better understand the species' abundance patterns and life cycle. Measures should be taken to prevent infiltration of wastewaters from the nearby town into the soil and underground habitats and to minimise the effects of the hydroelectric power station on the surrounding habitats.

##### Conservation actions

Conservation action type: In Place

Conservation actions: 1.1. Land/water protection - Site/area protection5.1.3. Law & policy - Legislation - Sub-national level

##### Conservation actions

Conservation action type: Needed

Conservation actions: 2.1. Land/water management - Site/area management4. Education & awareness

#### Conservation actions

Conservation action type: In Place

Conservation actions: 1.1. Land/water protection - Site/area protection5.1.3. Law & policy - Legislation - Sub-national level

#### Conservation actions

Conservation action type: Needed

Conservation actions: 2.1. Land/water management - Site/area management4. Education & awareness

#### Other

##### Use and trade

Use type: International

##### Ecosystem services

Ecosystem service type: Very important

##### Research needed

Research needed: 1.2. Research - Population size, distribution & trends1.3. Research - Life history & ecology2.1. Conservation Planning - Species Action/Recovery Plan3.1. Monitoring - Population trends3.4. Monitoring - Habitat trends

Justification for research needed: A sustainable conservation plan for the species is only possible if more information about population size, extent of distribution, ecology and life cycle is collected.

#### Use and trade

Use type: International

#### Ecosystem services

Ecosystem service type: Very important

#### Research needed

Research needed: 1.2. Research - Population size, distribution & trends1.3. Research - Life history & ecology2.1. Conservation Planning - Species Action/Recovery Plan3.1. Monitoring - Population trends3.4. Monitoring - Habitat trends

Justification for research needed: A sustainable conservation plan for the species is only possible if more information about population size, extent of distribution, ecology and life cycle is collected.

#### Viability analysis

### Domene darinkae

#### Species information

Scientific name: Domenedarinkae

Species authority: Magrini & Carotti 2019

Kingdom: Animalia

Phylum: Arthropoda

Class: Insecta

Order: Coleoptera

Family: Staphylinidae

Region for assessment: Europe

#### Geographic range

Biogeographic realm: Palearctic

Countries: Portugal

Map of records (Google Earth): Suppl. materials 1, 10

Basis of EOO and AOO: Known habitat extent

Basis (narrative): The extent of occurrence (EOO) and the maximum estimated area of occupancy (AOO) are both 4 km^2^.

Range description: *Domenedarinkae* is a cave-adapted rove beetle known from an abandoned mine in northern Portugal (Magrini and Carotti 2019).

#### New occurrences

#### Extent of occurrence

EOO (km2): 4

Trend: Unknown

Causes ceased?: Unknown

Causes understood?: Unknown

Causes reversible?: Unknown

Extreme fluctuations?: Unknown

#### Area of occupancy

Trend: Unknown

Causes ceased?: Unknown

Causes understood?: Unknown

Causes reversible?: Unknown

Extreme fluctuations?: Unknown

AOO (km2): 4

#### Locations

Number of locations: 1

Justification for number of locations: *Domenedarinkae* is known from a single horizontal artificial cave, Santa Isabel mine, located in the Marão Mountain chain in north Portugal (Magrini and Carotti 2019).

Trend: Unknown

Extreme fluctuations?: Unknown

#### Population

Number of individuals: Unknown

Trend: Unknown

Causes ceased?: Unknown

Causes understood?: Unknown

Causes reversible?: Unknown

Extreme fluctuations?: Unknown

Population Information (Narrative): Only one specimen of this species is known from a single location in northern Portugal (Magrini and Carotti 2019).

#### Subpopulations

Trend: Unknown

Extreme fluctuations?: Unknown

Severe fragmentation?: Unknown

#### Habitat

System: Terrestrial

Habitat specialist: Yes

Habitat (narrative): The only known specimen was collected in the rocky debris along the main tunnel of the Santa Isabel mine (Magrini and Carotti 2019). The geological matrix of this mine is quartzite with iron.

Trend in extent, area or quality?: Decline (inferred)

##### Habitat

Habitat importance: Major Importance

Habitats: 7.1. Caves and Subterranean Habitats (non-aquatic) - Caves

#### Habitat

Habitat importance: Major Importance

Habitats: 7.1. Caves and Subterranean Habitats (non-aquatic) - Caves

#### Ecology

Size: 6.93 mm (male holotype)

Generation length (yr): 1

Dependency of single sp?: Unknown

Ecology and traits (narrative): *Domenedarinkae* is included in the subgenus Lathromene. It is a predator and exhibits troglomorphisms, such as depigmentation, elongation of body and antennae and accentuated microphthalmia (Magrini and Carotti 2019).

#### Threats

##### Threats

Threat type: Past

Threats: 6.3. Human intrusions & disturbance - Work & other activities7.3. Natural system modifications - Other ecosystem modifications

#### Threats

Threat type: Past

Threats: 6.3. Human intrusions & disturbance - Work & other activities7.3. Natural system modifications - Other ecosystem modifications

#### Conservation

Justification for conservation actions: Undisturbed areas in the surface of the mine need to be defined and established.

##### Conservation actions

Conservation action type: Needed

Conservation actions: 1.1. Land/water protection - Site/area protection1.2. Land/water protection - Resource & habitat protection2.1. Land/water management - Site/area management4. Education & awareness

#### Conservation actions

Conservation action type: Needed

Conservation actions: 1.1. Land/water protection - Site/area protection1.2. Land/water protection - Resource & habitat protection2.1. Land/water management - Site/area management4. Education & awareness

#### Other

##### Use and trade

Use type: International

##### Ecosystem services

Ecosystem service type: Very important

##### Research needed

Research needed: 1.2. Research - Population size, distribution & trends1.3. Research - Life history & ecology2.2. Conservation Planning - Area-based Management Plan3.1. Monitoring - Population trends3.4. Monitoring - Habitat trends

Justification for research needed: Further information about population size, extent of distribution, ecology and life cycle is needed to better protect the species and the habitat. The mine, where the species was found, is an anthropogenic construction that clearly adversely affected the natural habitat of the species that should be the deep fissures and the mesovoid shallow substrate of the area. Therefore, it is recommended to sample these habitats in the area to understand the distribution of this species and to define new conservation priorities.

#### Use and trade

Use type: International

#### Ecosystem services

Ecosystem service type: Very important

#### Research needed

Research needed: 1.2. Research - Population size, distribution & trends1.3. Research - Life history & ecology2.2. Conservation Planning - Area-based Management Plan3.1. Monitoring - Population trends3.4. Monitoring - Habitat trends

Justification for research needed: Further information about population size, extent of distribution, ecology and life cycle is needed to better protect the species and the habitat. The mine, where the species was found, is an anthropogenic construction that clearly adversely affected the natural habitat of the species that should be the deep fissures and the mesovoid shallow substrate of the area. Therefore, it is recommended to sample these habitats in the area to understand the distribution of this species and to define new conservation priorities.

#### Viability analysis

## Discussion

The year 2021 is the International Year of Caves and Karst (http://iyck2021.org), an event organised by the International Union of Speleology to promote the awareness for the importance of caves and their habitats. Under this framework, a global initiative created the International Cave Animal of the Year (http://iyck2021.org/index.php/cave-animal-of-the-year) devoted to cave beetles. Within this initiative, different countries selected their own endemic species as a flag for advocating the conservation of subterranean ecosystems. Here, we offer information about the distribution (Suppl. material 1), habitat, species ecology, current threats and conservation measures for the nine cave-adapted beetles of continental Portugal. This information is essential to raise the awareness about the threats faced by subterranean ecosystems and to establish conservation measures needed specifically for each country.

Beetles are the most diverse insects in Portuguese caves (Reboleira et al. 2011a, Reboleira et al. 2017, Borges et al. 2019). Cave-adapted beetles from continental Portugal are highly endemic with a very reduced extent of occurrence (EOO) and area of occupancy (AOO). This occurs because they are all endemic from their correspondent karst massif unit or collected in artificial mines in granite or quartzite rock. Four of the known species are endemic to single caves, while the other five species can be found in more than one cave. The use of molecular methods may shed further light on the evolutionary relationships and species delimitation, especially for the genus *Trechus*, which shows a radiation in contiguous areas of karst massifis in central Portugal (Reboleira et al. 2010).

Most of the cave-adapted species of beetles are extremely rare, appearing only once or twice per decade of constant sampling. For example, *Iberoporuspluto*, the only groundwater-adapted (stygobiont) beetle from Portugal, was described, based on a female specimen and no further specimens have been found in a cave that has been constantly monitored for more than a decade. Its habitat, the Soprador do Carvalho Cave is under serious anthropogenic threats, such as groundwater contamination and touristic pressure (Ribera and Reboleira 2019).

Some of the major threats that cave-adapted species face are habitat destruction due to the intensive quarrying activity that occurs near the majority of the localities and the severe groundwater contamination caused by the lack of proper wastewater treatment in most villages of the central Portugal karst massifs. Some of these threats have been identified previously by Reboleira et al. 2011b, but no specific conservation measures have been taken hitherto. The infiltration of contaminants and fertilisers originating from agricultural practices and industry on the surface, also pose major threats to the integrity of subterranean organisms (Castaño-Sánchez et al. 2020). Therefore, it is urgent to generate ecotoxicological data on the sensitivity of cave-adapted beetles to understand if the pernicious impacts that have been already evaluated for some endemic groundwater-adapted species in Portugal (Reboleira et al. 2013b) are also impacting the terrestrial cave species.

In order to create protection strategies for cave-adapted species in continental Portugal, it is necessary to improve our knowledge about their population size, extent of distribution, ecology and life cycle. We hope this contribution may help to support decision-making on territory planning and to establish conservation measures for these highly endemic species. These will act as umbrella species for the conservation of other cave-adapted species that share the same subterranean habitats.

Discussion

## Supplementary Material

2A6A5D0E-3848-5F44-878A-A57171133B7B 10.3897/BDJ.9.e67426.suppl1 Supplementary material 1 Distribution of cave-adapted beetles in continental Portugal. Data type Species distribution map Brief description 1: *Trechusmachadoi* (blue circle); 2: *T.gamae* (yellow circle); 3:*T.lunai* (pink circle); 4: *T.tatai* (red circle); 5: *Iberoporuspluto* (blue star); 6: *Domenelusitanica* (yellow diamond); 7: *D.viriatoi* (pink diamond); 8: *D.darinkae* (blue diamond); and 9: *Speonemadusalgarvensis* (pink triangle). (A) Detail of northern distribution, (B) Detail of central distribution and (C) Detail of southern distribution. In green are protected areas. File: oo_528602.tif https://binary.pensoft.net/file/528602 A.S.P.S. Reboleira, R.P. Eusébio

0F7E68E8-AAD0-50EE-B880-0CEFA740227D 10.3897/BDJ.9.e67426.suppl2 Supplementary material 2 Distribution of cave-adapted beetle *Iberoporuspluto*. Data type Species distribution map Brief description *Iberoporuspluto* distribution: Soprador do Carvalho Cave, Penela, Coimbra District. File: oo_560421.tif https://binary.pensoft.net/file/560421 A.S.P.S. Reboleira, R.P. Eusébio

D7DABAF2-3B19-5E93-BD25-C30939739EB6 10.3897/BDJ.9.e67426.suppl3 Supplementary material 3 Distribution of cave-adapted beetle *Trechusmachadoi*. Data type Species distribution map Brief description *Trechusmachadoi* distribution: Alcobertas Cave and mesovoid shallow substratum, Rio Maior. File: oo_560428.tif https://binary.pensoft.net/file/560428 A.S.P.S. Reboleira, R.P.Eusébio

F33828A8-DED8-539A-8ADB-E01A34FF86B1 10.3897/BDJ.9.e67426.suppl4 Supplementary material 4 Distribution of cave-adapted beetle *Trechusgamae*. Data type Species distribution map Brief description *Trechusgamae* distribution: (1) Algar da Arroteia Cave; (2) Fórnea (MSS); (3) Algar do Ladoeiro Cave; (4) Algar de Marradinhas II Cave; (5) Algar do Pena Cave; and (6) Algar das Gralhas VII Cave. All caves and MSS are located in the Santo António Plateau, the central subunit of the Estremenho karst massif. (A) Detail of distribution. File: oo_560430.tif https://binary.pensoft.net/file/560430 A.S.P.S. Reboleira, R.P. Eusébio

50F0176B-32A1-5094-B243-043C8F72D142 10.3897/BDJ.9.e67426.suppl5 Supplementary material 5 Distribution of cave-adapted beetle *Trechuslunai*. Data type Species distribution map Brief description *Trechuslunai* distribution: (1) Contenda and Moinhos Velhos Cave system; and (2) Almonda Cave, both located in the Estremenho karst massif. File: oo_560431.tif https://binary.pensoft.net/file/560431 A.S.P.S. Reboleira, R.P. Eusébio

8999FE89-5F39-5920-80EA-13A029878CF1 10.3897/BDJ.9.e67426.suppl6 Supplementary material 6 Distribution of cave-adapted beetle *Trechustatai*. Data type Species distribution map Brief description *Trechustatai* distribution: Algar do Javali Cave, Montejunto karst massif. File: oo_560436.tif https://binary.pensoft.net/file/560436 A.S.P.S. Reboleira, R.P. Eusébio

3044C8C3-6105-508F-AB09-06C452FC8637 10.3897/BDJ.9.e67426.suppl7 Supplementary material 7 Distribution of cave-adapted beetle *Speonemadusalgarvensis*. Data type Species distribution map Brief description *Speonemadusalgarvensis* distribution: (1) Algarão do Remexido Cave; (2) Vale Telheiro Cave; and (3) Senhora cavea, all located in the Algarve karst massif. File: oo_560437.tif https://binary.pensoft.net/file/560437 A.S.P.S. Reboleira, R.P. Eusébio

2C303E65-CCAC-500D-B815-0FAE2AEA3BFE 10.3897/BDJ.9.e67426.suppl8 Supplementary material 8 Distribution of cave-adapted beetle *Domenelusitanica*. Data type Species distribution map Brief description *Domenelusitanica* distribution: Cerâmica Cave, Sicó karst area. File: oo_560438.tif https://binary.pensoft.net/file/560438 A.S.P.S. Reboleira, R.P. Eusébio

081BE9A5-9F66-5DA2-8DF0-7B8416C9E0E1 10.3897/BDJ.9.e67426.suppl9 Supplementary material 9 Distribution of cave-adapted beetle *Domeneviriatoi*. Data type Species distribution map Brief description *Domeneviriatoi* distribution: Buraco da Moura Cave, Estrela Mountain chain. File: oo_560439.tif https://binary.pensoft.net/file/560439 A.S.P.S. Reboleira, R.P. Eusébio

6A5B9DF4-9ACF-5EC2-B7F1-3228812F5DEC 10.3897/BDJ.9.e67426.suppl10 Supplementary material 10 Distribution of cave-adapted beetle *Domenedarinkae*. Data type Species distribution map Brief description *Domenedarinkae* distribution: Santa Isabel mine, Marão Mountain chain. File: oo_560442.tif https://binary.pensoft.net/file/560442 A.S.P.S. Reboleira, R.P. Eusébio

## Hosting institution

Natural History Museum of Denmark
