# Supplementary figures and images for: Cave-adapted beetles from continental Portugal
Source: Biodivers Data J. 2021 Aug 20;9:e67426. doi: 10.3897/BDJ.9.e67426 (PMC8397697; doi:10.3897/BDJ.9.e67426)

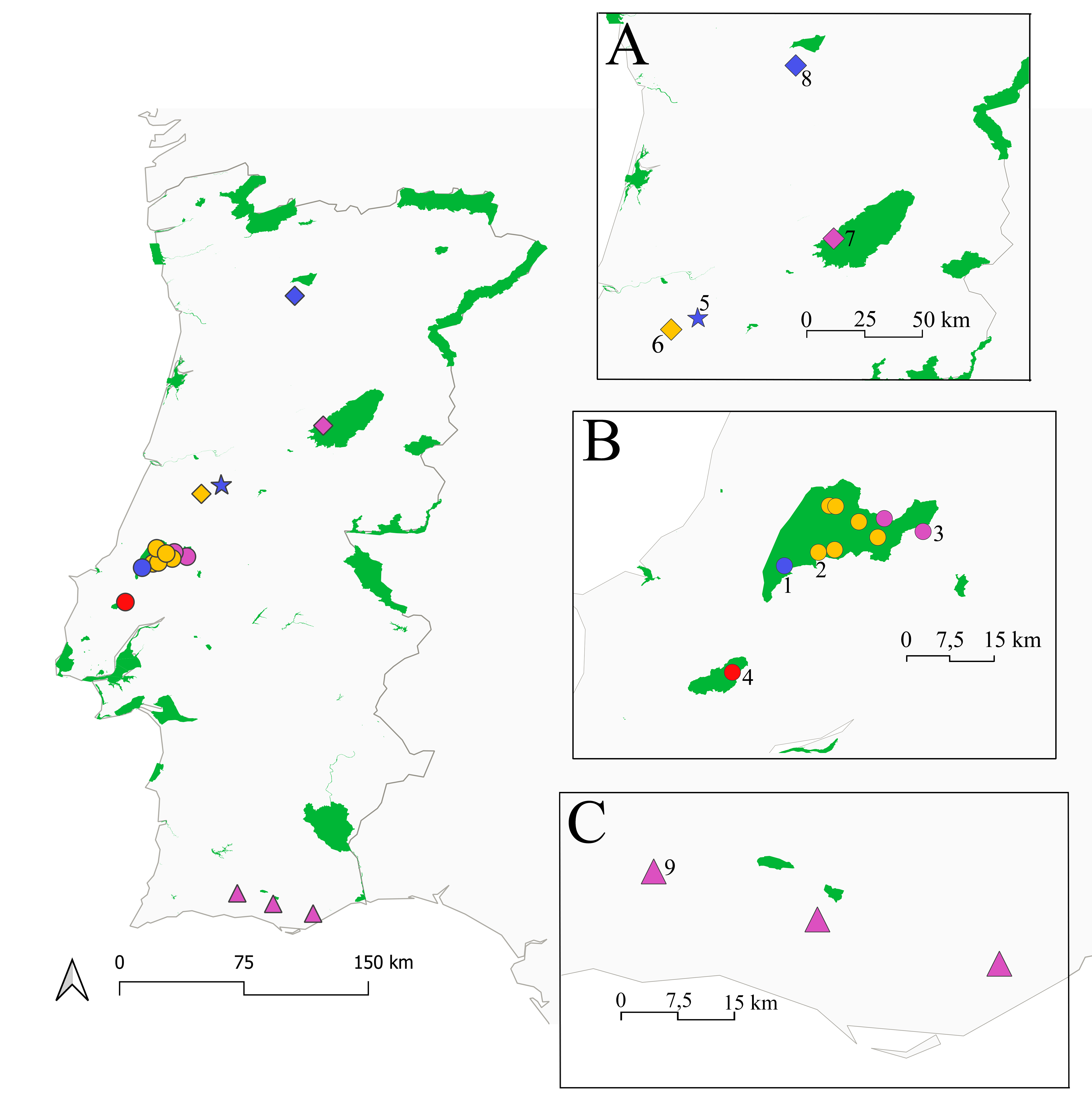

Supplement: Supplementary material 1 — Distribution of cave-adapted beetles in continental Portugal. [file bdj-09-e67426-s001.tif]

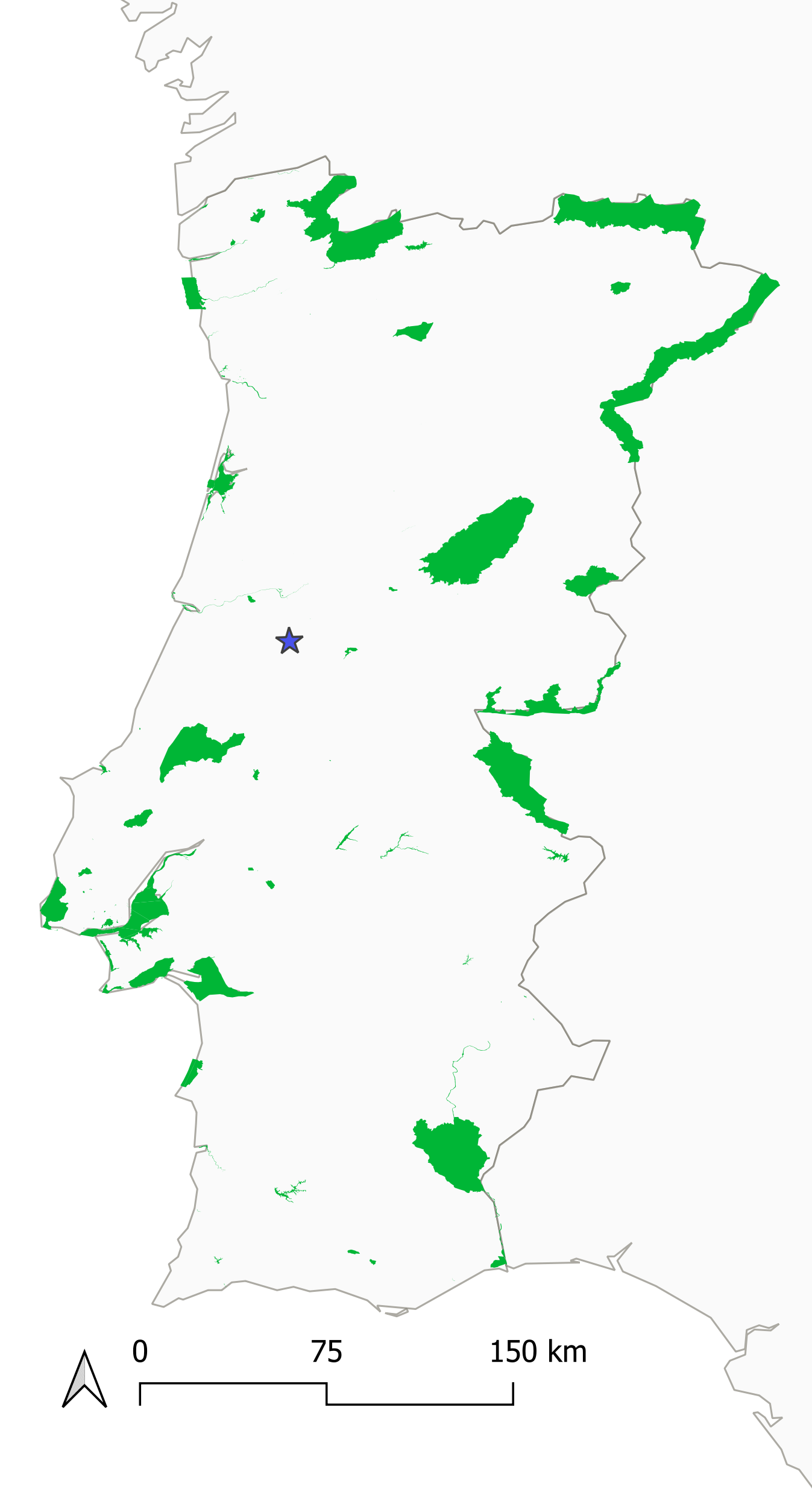

Supplement: Supplementary material 2 — Distribution of cave-adapted beetle Iberoporuspluto. [file bdj-09-e67426-s002.tif]

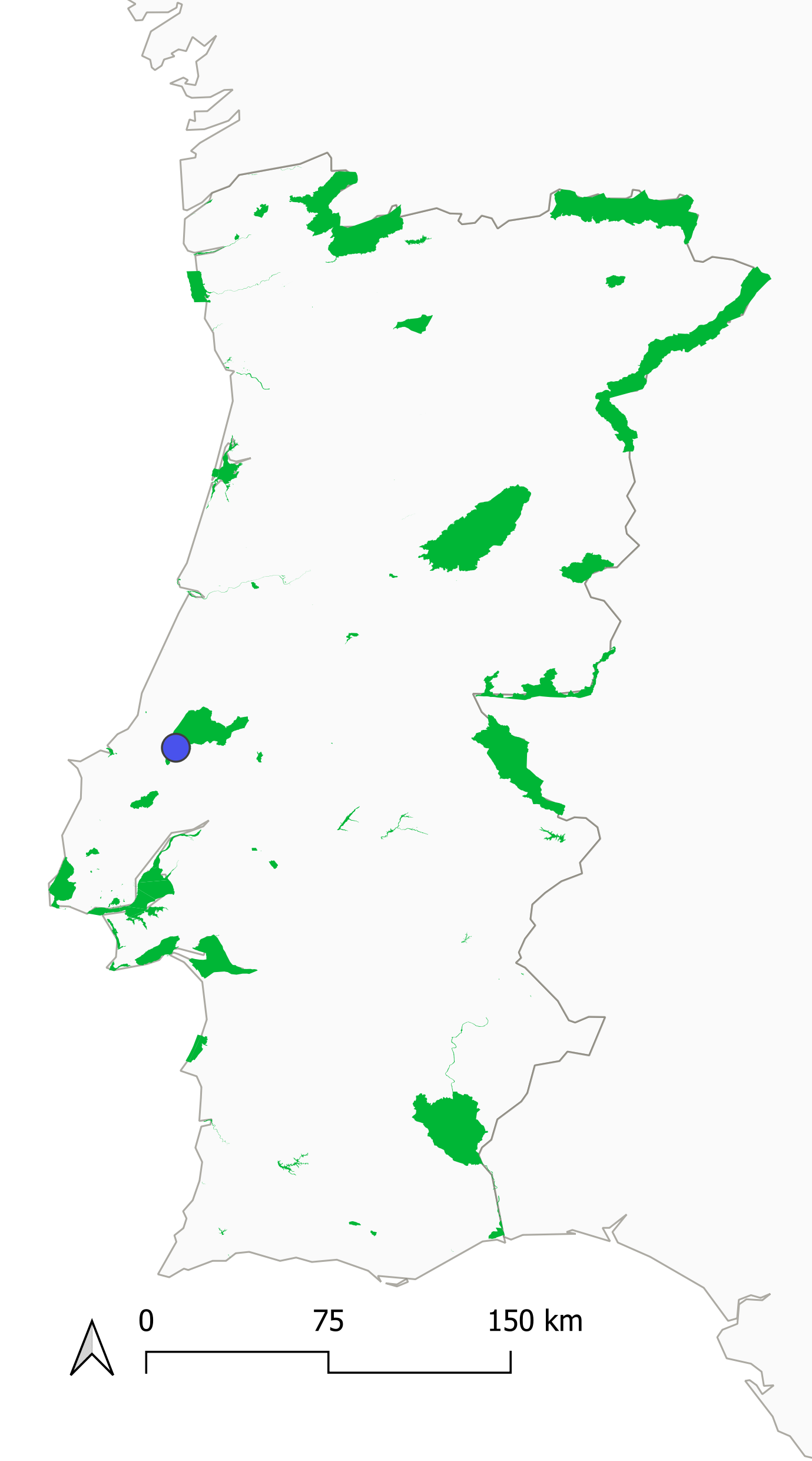

Supplement: Supplementary material 3 — Distribution of cave-adapted beetle Trechusmachadoi. [file bdj-09-e67426-s003.tif]

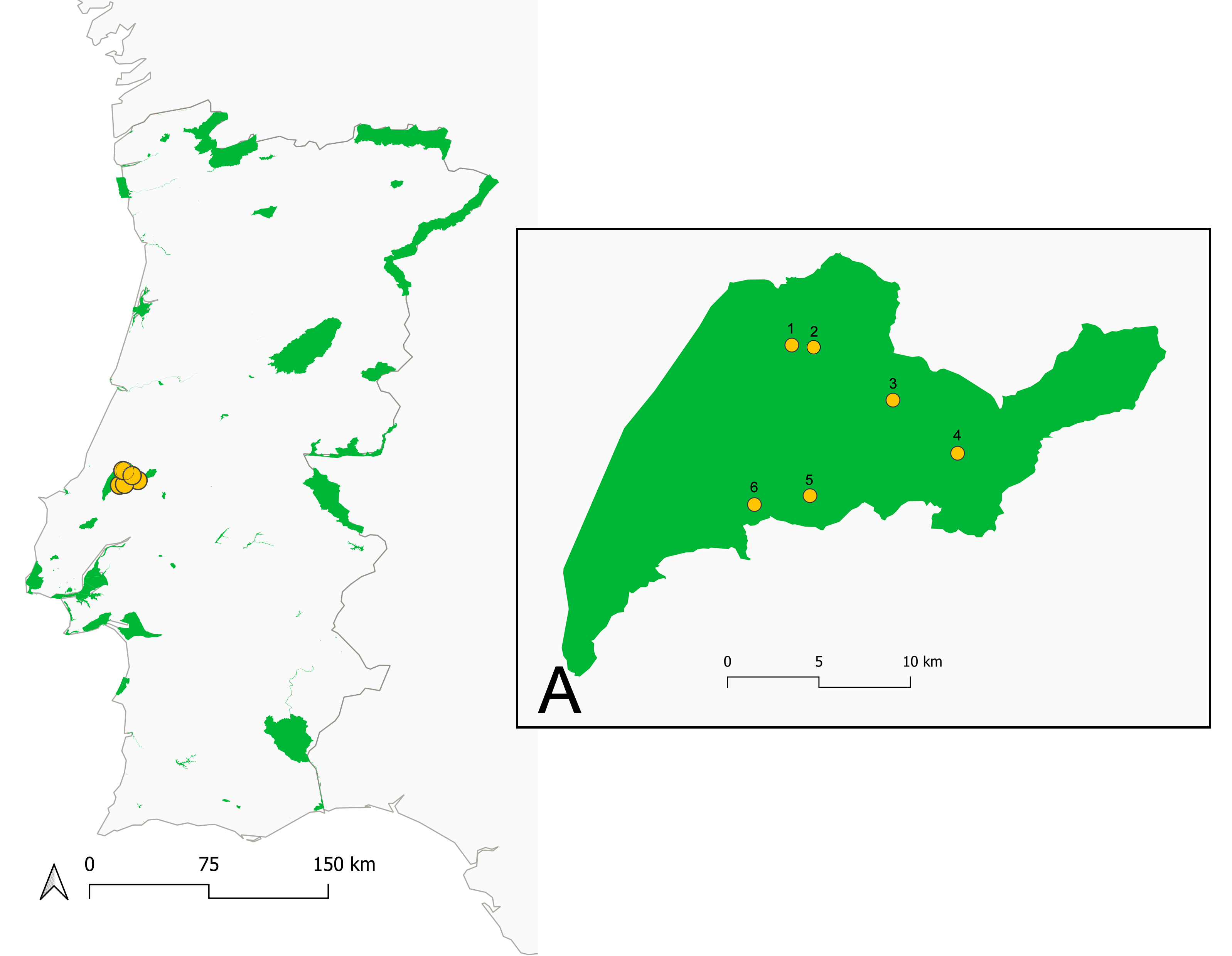

Supplement: Supplementary material 4 — Distribution of cave-adapted beetle Trechusgamae. [file bdj-09-e67426-s004.tif]

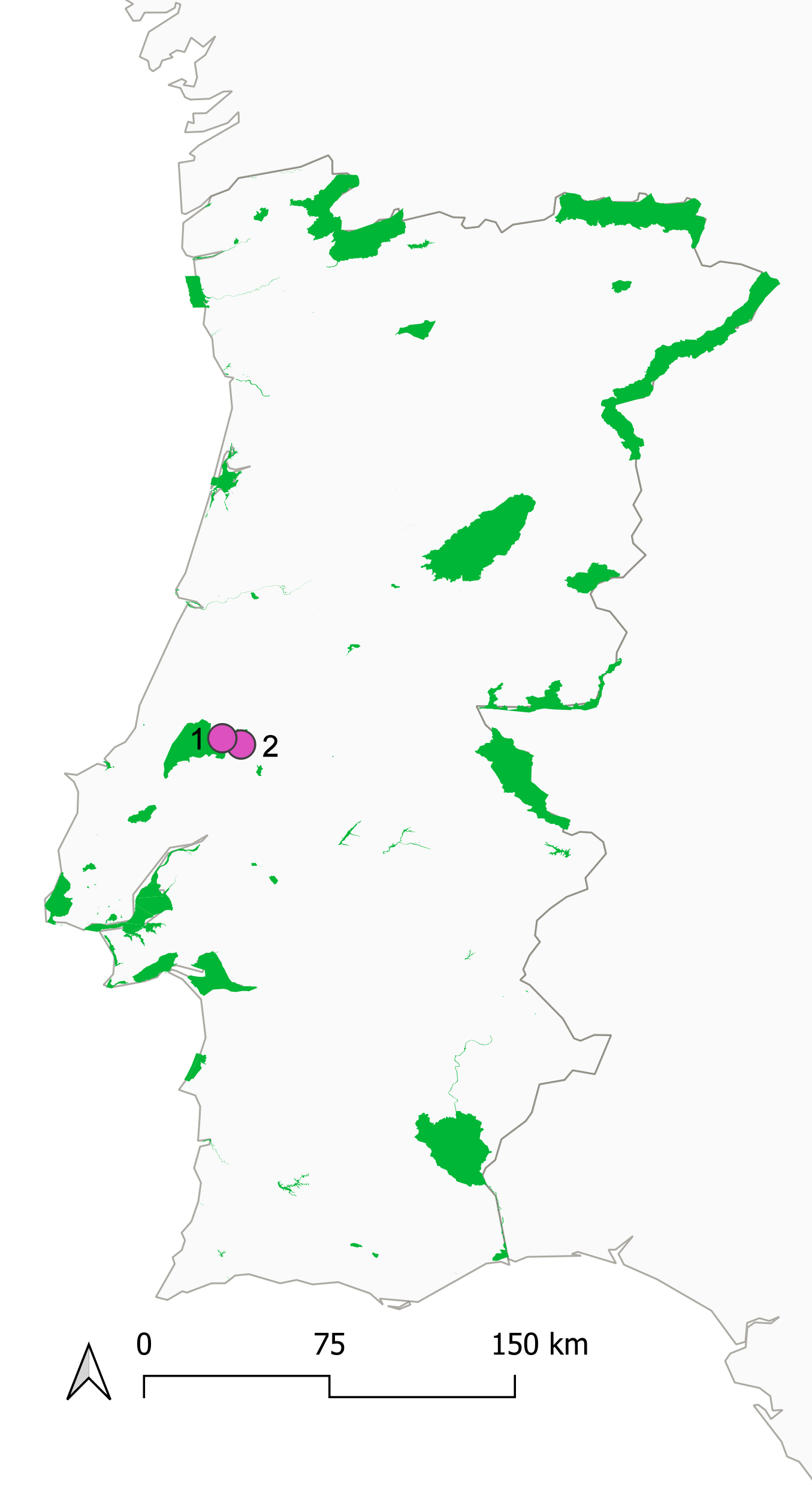

Supplement: Supplementary material 5 — Distribution of cave-adapted beetle Trechuslunai. [file bdj-09-e67426-s005.tif]

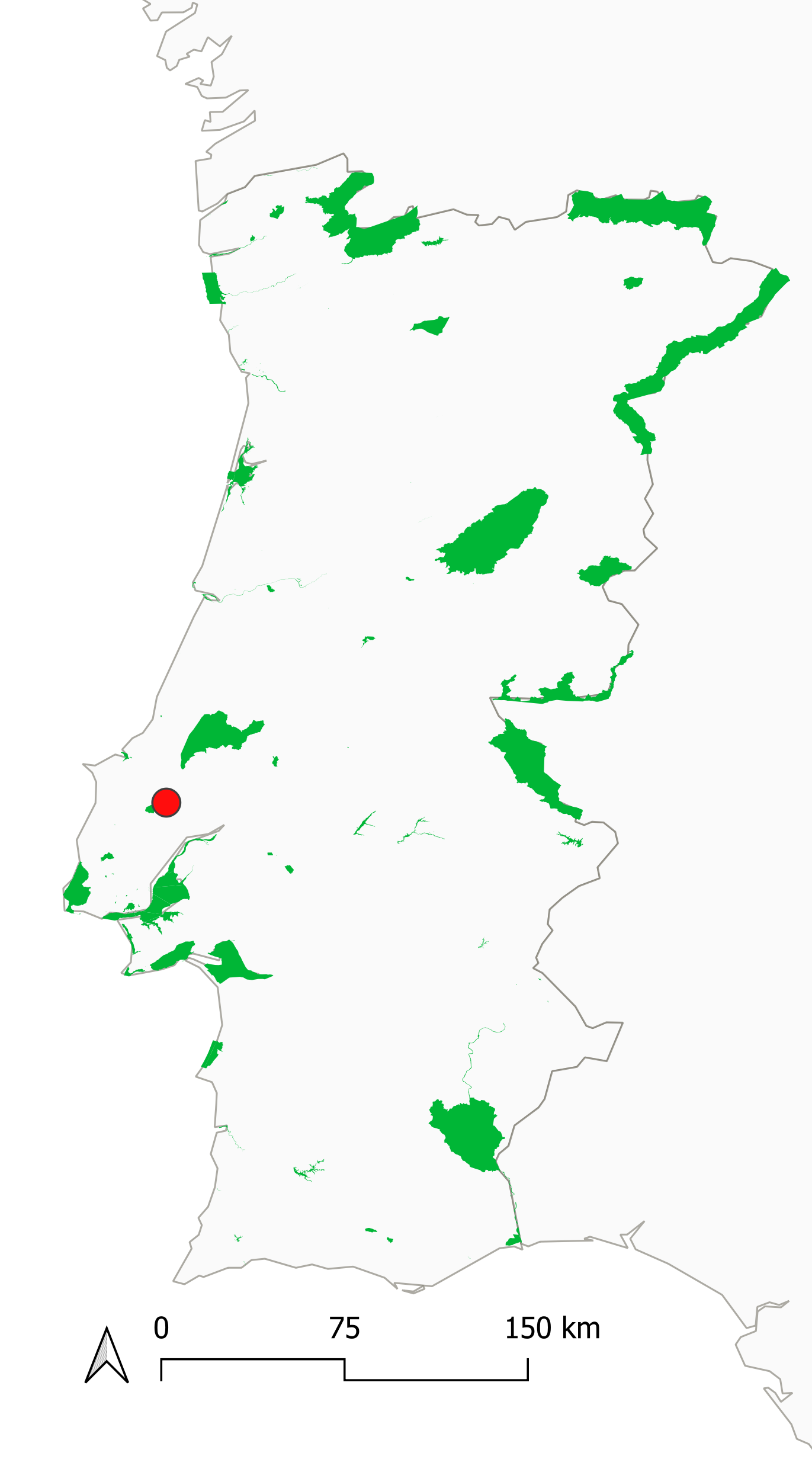

Supplement: Supplementary material 6 — Distribution of cave-adapted beetle Trechustatai. [file bdj-09-e67426-s006.tif]

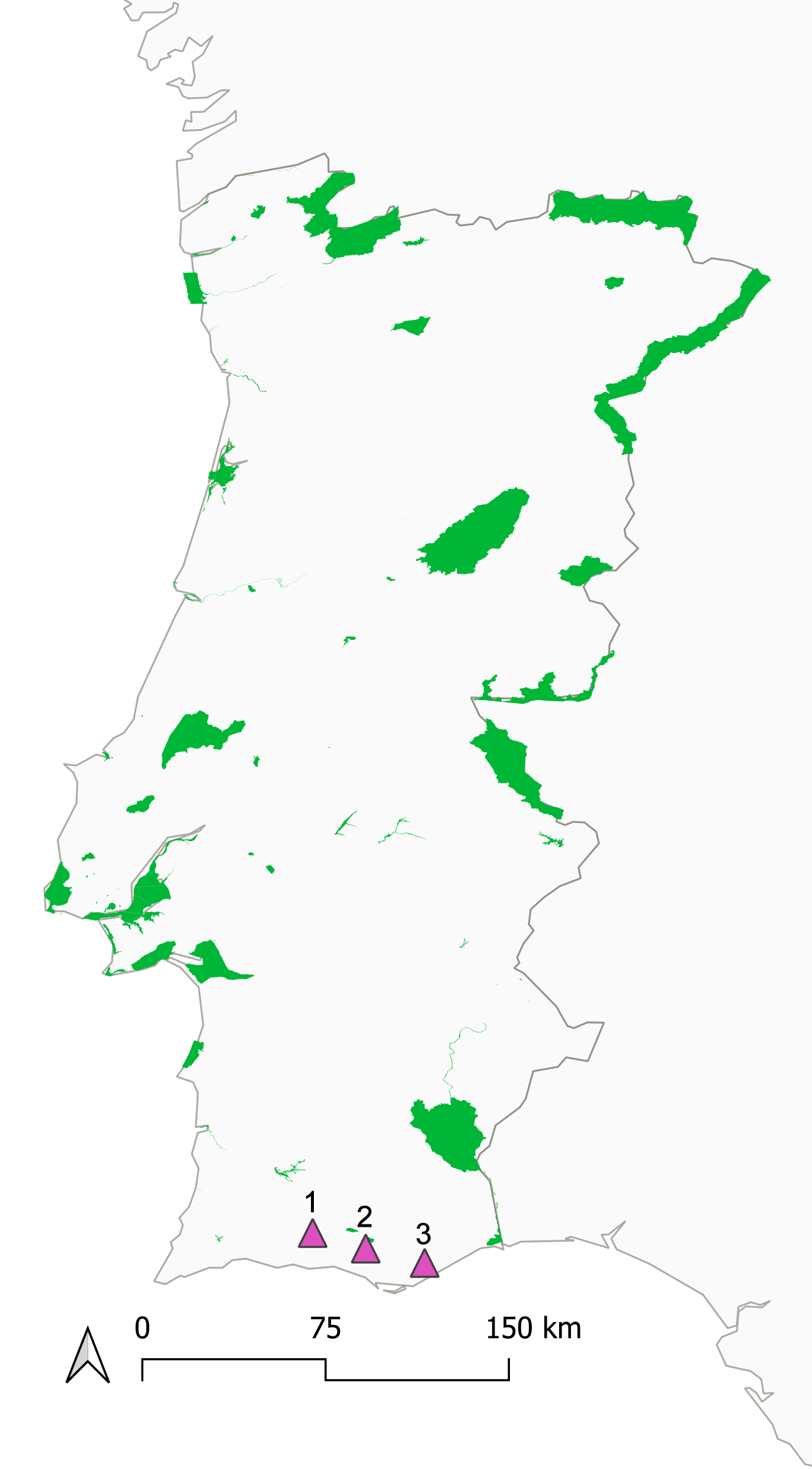

Supplement: Supplementary material 7 — Distribution of cave-adapted beetle Speonemadusalgarvensis. [file bdj-09-e67426-s007.tif]

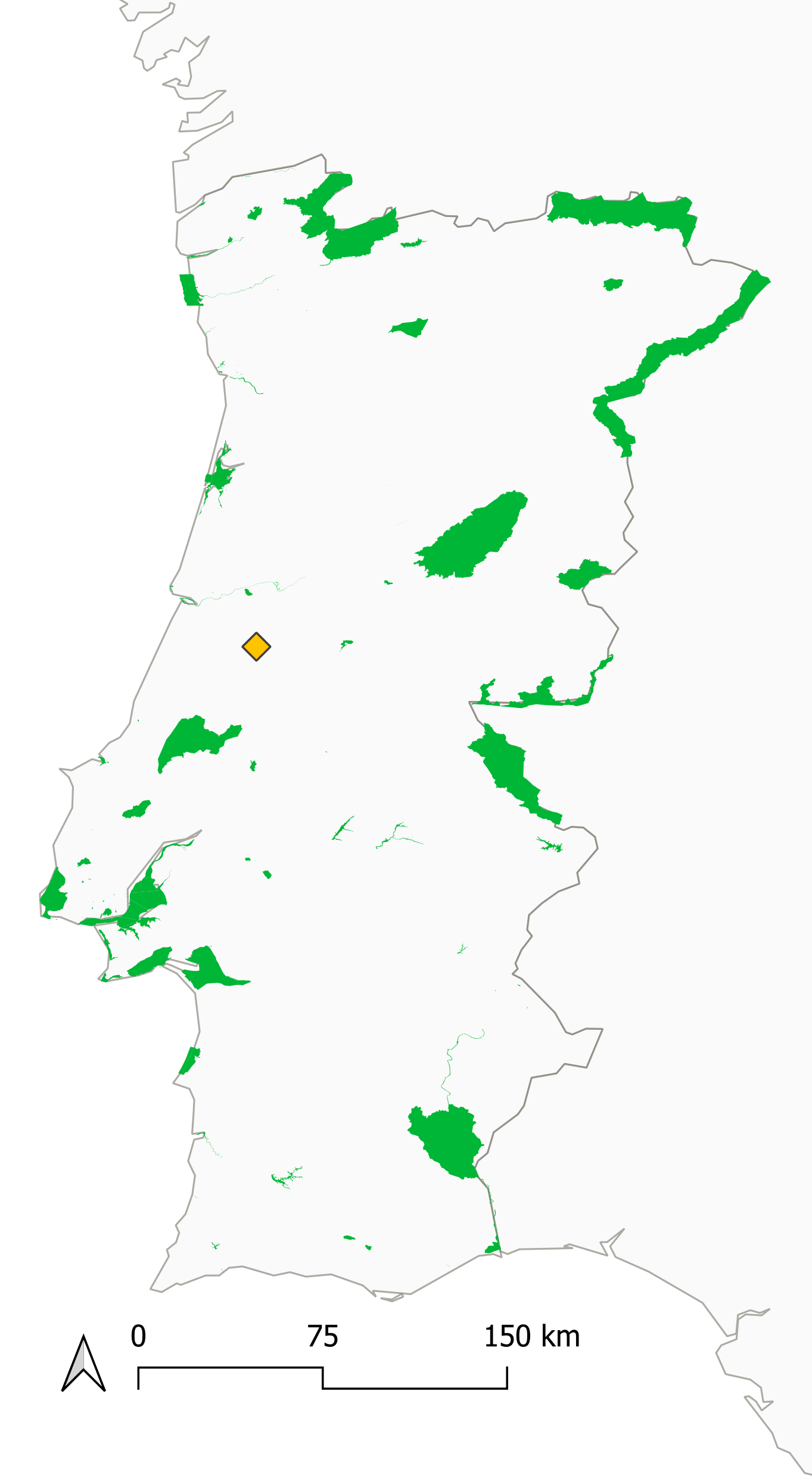

Supplement: Supplementary material 8 — Distribution of cave-adapted beetle Domenelusitanica. [file bdj-09-e67426-s008.tif]

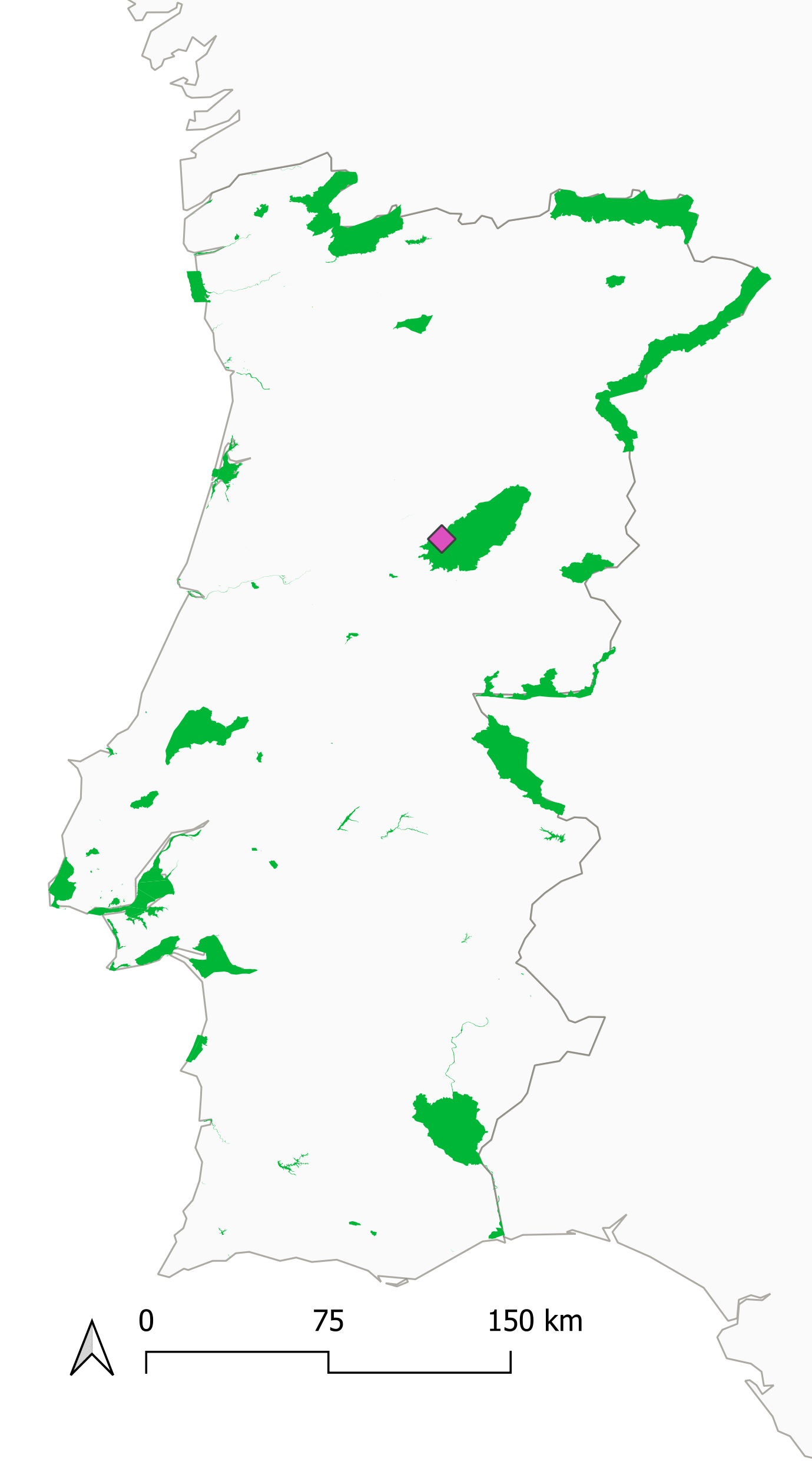

Supplement: Supplementary material 9 — Distribution of cave-adapted beetle Domeneviriatoi. [file bdj-09-e67426-s009.tif]

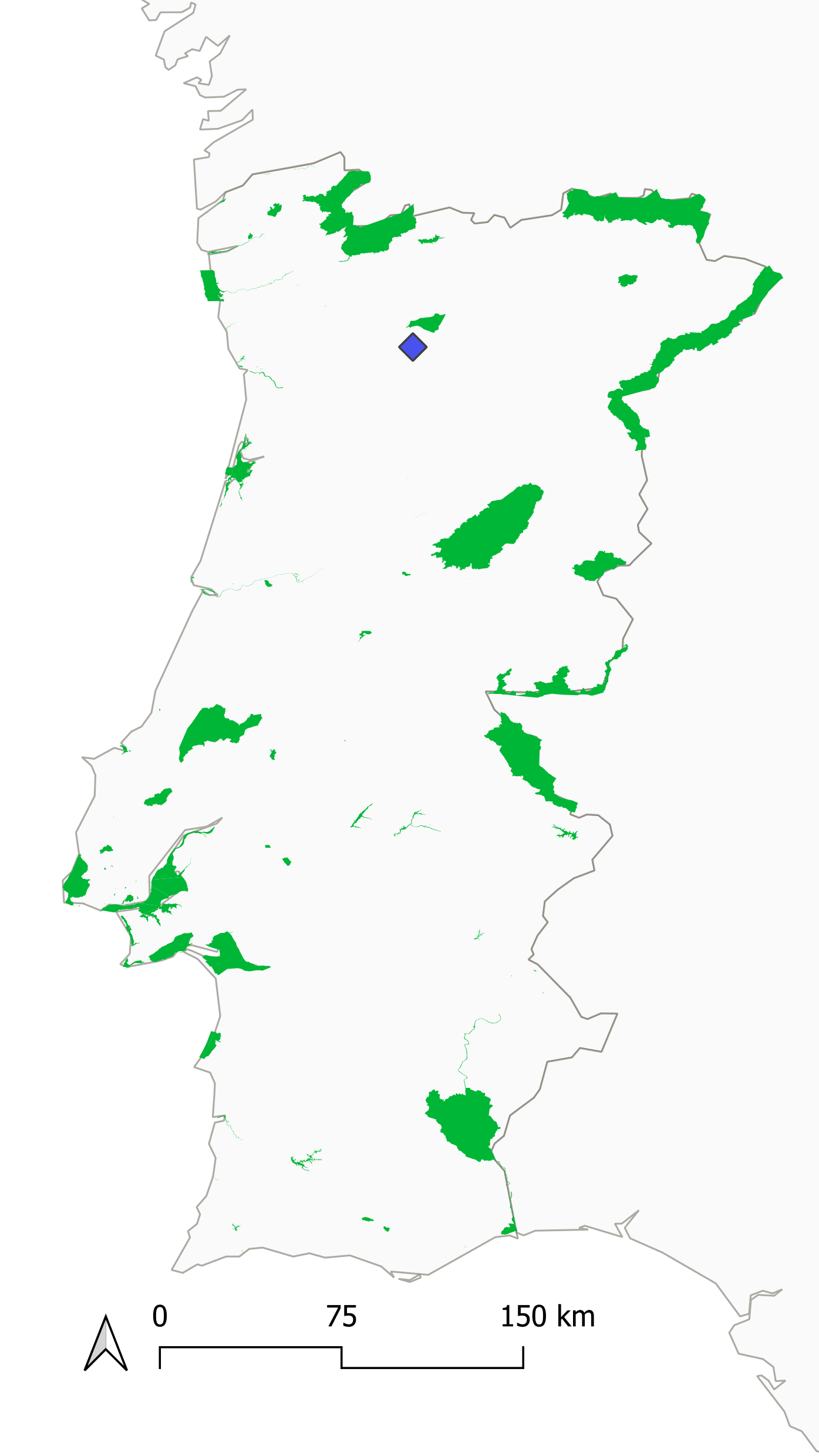

Supplement: Supplementary material 10 — Distribution of cave-adapted beetle Domenedarinkae. [file bdj-09-e67426-s010.tif]
